# Rethinking Discount Regularization: New Interpretations, Unintended Consequences, and Solutions for Regularization in Reinforcement Learning

**Published:** 2024

**Authors:** Sarah Rathnam, Sonali Parbhoo, Siddharth Swaroop, Weiwei Pan, Susan A. Murphy, Finale Doshi-Velez

**Affiliations:** John A. Paulson School of Engineering and Applied Sciences, Harvard University, Cambridge, MA 02138 USA; Imperial College London, London SW7 2BX, UK; John A. Paulson School of Engineering and Applied Sciences, Harvard University, Cambridge, MA 02138 USA; John A. Paulson School of Engineering and Applied Sciences, Harvard University, Cambridge, MA 02138 USA; John A. Paulson School of Engineering and Applied Sciences, Harvard University, Cambridge, MA 02138 USA; John A. Paulson School of Engineering and Applied Sciences, Harvard University, Cambridge, MA 02138 USA

**Keywords:** reinforcement learning, regularization, certainty equivalence, discount factor, Markov decision process

## Abstract

Discount regularization, using a shorter planning horizon when calculating the optimal policy, is a popular choice to avoid overfitting when faced with sparse or noisy data. It is commonly interpreted as de-emphasizing or ignoring delayed effects. In this paper, we prove two alternative views of discount regularization that expose unintended consequences and motivate novel regularization methods. In model-based RL, planning under a lower discount factor acts like a prior with stronger regularization on state-action pairs with more transition data. This leads to poor performance when the transition matrix is estimated from data sets with uneven amounts of data across state-action pairs. In model-free RL, discount regularization equates to planning using a weighted average Bellman update, where the agent plans as if the values of all state-action pairs are closer than implied by the data. Our equivalence theorems motivate simple methods that generalize discount regularization by setting parameters locally for individual state-action pairs rather than globally. We demonstrate the failures of discount regularization and how we remedy them using our state-action-specific methods across empirical examples with both tabular and continuous state spaces.

## 1 Introduction

In reinforcement learning (RL), planning under a shorter horizon is a common form of regularization with a straightforward interpretation: restricting policy class complexity by optimizing for shorter term rewards (Jiang et al., 2015). In the most extreme case, planning with a discount factor of zero results in a contextual bandit algorithm. Using a reduced or zero discount factor for planning is common in real-world applications such as mobile health (Liao et al., 2020; Trella et al., 2022), medicine (Oh et al., 2022; Awasthi et al., 2022; Durand et al., 2018), and education (Cai et al., 2021; Qi et al., 2018), particularly in low-data settings.

In this paper, we demonstrate the equivalence between discount regularization and other common regularization techniques. These connections provide a deeper understanding of discount regularization that reveals its limitations. We first prove that in model-based tabular RL, discount regularization produces the same optimal policy as averaging the transition matrix with a regularization matrix that is the same for all states and actions. In this view, discount regularization forces the agent to plan as if the distribution over next state is more similar to one another across different states and actions than was observed in the data. This can also be viewed in terms of a prior on the transition matrix. We prove an analogous result for model-free RL: discount regularization produces the same optimal policy as a modified Q-function update rule of a specific weighted-average form. In this form, we see that discount regularization acts similarly to a penalty on the value function, forcing the state-action values to be more similar. Finally, we demonstrate mathematically that discount regularization approximates a λ-return.

Reframing discount regularization in these ways exposes unintended consequences. One consequence is that the magnitude of the prior implied by discount regularization is higher for state-action pairs with more transition observations in the data and vice versa. This is generally not desirable as we want stronger regularization on state-action pairs that we have observed less, and to rely on the data in those that we have observed more. Another negative effect is that the implied prior has the same transition distribution for all state-action pairs. This is inappropriate in many contexts, where it is better to replace this implicit prior with an informed prior that reflects knowledge about the environment.

Our equivalence theorems motivate model-free and model-based offline regularization methods that offer solutions to the problems exposed above. In the model-based setting, we apply *certainty-equivalence RL*, where the agent takes the estimated model as true when calculating the optimal policy (Goodwin and Sin, 1984). We take a simple estimate of the transition model (MLE for a tabular state space or kernel regression if continuous) and regularize the estimate to use within simple RL methods. Our methods tailor regularization to the task at hand, which includes both the data set and the environment. To mitigate the issue of inconsistent prior magnitudes in data sets with uneven exploration, we derive a state-action specific formula for the regularization parameter. The method we use to derive this parameter can be adapted to other priors to match the transition dynamics of the environment.

Finally, we demonstrate our results empirically. First, we confirm our equivalence theorems in a tabular setting. We then demonstrate that a uniform prior with fixed magnitude across state-action pairs outperforms discount regularization across environments. We compare our model-based and model-free state-action-specific regularization methods to each other as well as to regularization with a fixed global parameter. Then, we demonstrate that our model-based method extends successfully to a continuous state space.

This paper extends previous work published in Rathnam et al. (2023). The major extensions are (1) a model-free analysis that connects discount regularization to a penalized Q-function, (2) analysis of how discount regularization approximates a truncated lambda-return, (3) introduction, analysis and simulations of a novel model-free regularization method, including its relationship to pessimism, and (4) application of novel regularization methods to a continuous state space.

## 2 Related Works

### Discount Regularization.

Jiang et al. (2015) demonstrate that the optimal policy generated using a “planning discount factor” that is shorter than the true discount factor of the MDP often outperforms the policy learned using the true discount factor when both policies are evaluated in the true environment (using the true discount factor). They prove that using a lower planning discount factor to calculate the optimal policy controls model complexity by restricting the number of possible policies considered, thereby avoiding overfitting. They further demonstrate that the benefit of a lower planning discount factor is increasingly pronounced in cases where the model is estimated from a smaller data set. Amit et al. (2020) refer to this concept as “discount regularization,” a term which we use here. Unlike these works, we provide means to connect discount regularization with placing a prior on the transition matrix.

Previous works also discuss the limitations of a fixed discount factor and present approaches for more flexible discounting, for example state-dependent (Wei and Guo, 2011; Yoshida et al., 2013), state-action-dependent (Pitis, 2019), and transition-based discounting (White, 2017). We add to this work by demonstrating that discount regularization carries implicit assumptions of equal transition distributions for all state-action pairs and stronger regularization on those with more transition data.

### Bayesian RL.

While a Bayesian prior encodes expert knowledge, information from previous studies, or other outside information, we can also view a prior as a form of regularization since it forces the model not to overfit when data is limited (Poggio and Girosi, 1990; Ghavamzadeh et al., 2015). This is a flexible tool that allows us to regularize in a way that matches our prior knowledge and beliefs about the environment. In model-based Bayesian RL, the problem is often framed as a Bayes-Adaptive MDP (BAMDP), an MDP where the states are replaced by “hyperstates” that reflect the original state space combined with the posterior parameters of the transition function (Duff, 2002). In general, Bayesian RL algorithms do not explicitly address planner overfitting; rather they incorporate the probability distribution over models, causing the planner not to overfit to an uncertain model. For example, model-based Bayesian RL methods draw sample models from the posterior (Asmuth et al., 2012), sample hyperstates (Poupart et al., 2006), or apply an exploration bonus based on the amount of data (Kolter and Ng, 2009a) or based on the variance of the parameters (Sorg et al., 2012). The BAMDP framework can also be extended to the case of partial observability (Ross et al., 2007, 2011). In this paper, we discuss planning using the posterior mean of the transition matrix under a Dirichlet prior as a regularized form of the transition matrix, which is a common choice in model-based RL, e.g. Vlassis et al. (2012); O’Donoghue et al. (2020). In contrast to Bayesian methods, the methods that we propose regularize the model to get a point estimate which can be used directly in simple non-Bayesian RL methods.

### Penalized Value Function.

In the model-free setting, we relate discount regularization to a modified value function. Previous RL regularization methods work by adding a bonus or penalty to the value function. For example, an $L_{1}$ (Kolter and Ng, 2009b; Liu et al., 2012; Ghavamzadeh et al., 2011) or $L_{2}$ (Farebrother et al., 2018; Cobbe et al., 2019) penalty is commonly added to the value function. Entropy regularization can function by adding an entropy bonus to the gradient of the Q-function (O’Donoghue et al., 2016) or the reward (Nachum et al., 2017). Another use of a value function bonus is to encourage exploration by adding a bonus to the value of unseen states (Kolter and Ng, 2009a). Conversely, offline methods often employ pessimism penalize the value of states and actions not well-explored in the data. We analyze our method in the context of point-wise pessimism Jin et al. (2021) and Bellman-consistent pessimism Xie et al. (2021) in Sec. 5.2.3.

### Connecting Regularization Methods.

Several previous works have established connections between regularization methods, as we do here. For example, Wu et al. (2019) introduce a framework that connects a penalty on value function with policy regularization, Neu et al. (2017) connect entropy-regularized algorithms, and Li et al. (2006) present a unified view of state aggregation schemes. Liu et al. (2019) empirically compare a wider range of regularizers. More recently, Eysenbach et al. (2023) prove that one-step RL and critic regularization methods result in the same policy under certain assumptions. Most similar to our work, Amit et al. (2020) connect discount regularization with $L_{2}$ regularization in TD learning. We discuss the connection to this work in Section 4.3.

## 3 Background and Notation

### Markov Decision Process.

We consider a finite, discrete Markov decision process (MDP). An MDP M is characterized by $\langle S,A,R,T,\gamma \rangle$, defined as follows. S: State space of size $N_{s}$. A: Action space of size $N_{a}$. $R\left(s,a\right)$: Reward, as a function of state s and action a. $T\left(s,a,\cdot \right)$: Transition function, mapping each state-action pair to a probability distribution over successor states, assumed unknown and estimated from the data. γ: True discount factor for the MDP, 0≤γ<1, under which a policy π is evaluated. We also use the following notation: $\gamma_{p}$: Planning discount factor $0\leq \gamma_{p}<\gamma$. $\gamma_{p}$ is not used to evaluate the policy; it is used for planning only, where replacing γ with $\gamma_{p}$ serves as a regularizer ; $c_{i,j,k}$: count of transition observations in data set starting at state $s_{i}$, taking action $a_{j}$ and transitioning to state $s_{k}$.

### Certainty Equivalence.

The model-based portion of our analysis is in the context of certainty-equivalence RL. Certainty equivalence is a useful approach to model-based RL where the agent takes the estimated model as accurate when finding the optimal policy. It separates the estimation of the model from the policy optimization (Goodwin and Sin, 1984). The maximum likelihood estimate (MLE) is a natural choice for the model estimate, however maximum likelihood solutions can overfit, particularly in the case of small data sets (Murphy, 2012). Often, a better policy is obtained by regularizing the MDP before learning the certainty-equivalence policy.

## 4 Alternative Views of Discount Regularization and Connections to Other Regularization Methods

Discount regularization is a simple concept—the agent finds an optimal policy using a shorter horizon than the environment’s true horizon γ—yet analyzing its relationship to other methods provides new insights on regularization. In the sections that follow, we will demonstrate how discount regularization relates to different methods and interpretations: a weighted average T, a Bayesian prior on T, a penalized Q-function, and λ-returns.

### 4.1 Discount Regularization as a Weighted Average Transition Matrix

We begin with a reframing of discount regularization as a weighted average transition matrix. This form illustrates the classic view of discounting as partial termination and motivates our first equivalence theorem.

#### 4.1.1 Discount Regularization as Partial Termination

First, we show that discount regularization is mathematically equivalent to replacing the transition matrix with the weighted average between that transition matrix and a matrix of zeros. The form is unusual as the matrix of zeros is not a transition matrix, however it gives intuition on discount regularization and motivates the equivalence theorem and Bayesian formulation that follow.

To cast discount regularization in certainty-equivalence RL as a weighted average transition matrix, consider the Bellman equation for the value of each state under policy π, $V^{\pi }=R_{\pi }+\gamma T_{\pi }V^{\pi }$, where the vector $V^{\pi }$ is the value of each state, $R_{\pi }$ is the vector of rewards, and $T_{\pi }$ is the transition matrix, all under policy π. Let $\gamma_{p}<\gamma$ be the planning discount factor, the lower discount factor used for regularization when calculating the certainty-equivalence policy. (This policy will be evaluated under the true discount factor γ.) Then we have the Bellman equation $V^{\pi }=R_{\pi }+\gamma_{p}T_{\pi }V^{\pi }$. We rewrite the product $\gamma_{p}T_{\pi }$ from the Bellman equation as the product of true discount factor γ and a weighted average matrix: $\gamma_{p}T_{\pi }=\gamma \left[\left(1-\epsilon \right)T_{\pi }+\epsilon T_{\text{zeros}}\right]$, where $T_{\text{zeros}}$ is an appropriately sized matrix of zeros and $\epsilon =\frac{\gamma -\gamma_{p}}{\gamma }$.

Using this insight, when estimating the transition matrix from data, we can use the following weighted average transition matrix and the true discount factor γ for planning in place of the MLE transition matrix and lower discount factor $\gamma_{p}$.^1^

(1) $${\hat{T}}_{\begin{array}{c} \text{disc}\\ \text{reg} \end{array}}\left(s_{i},a_{j},\cdot \right)=\left(1-\epsilon \right){\hat{T}}_{\text{MLE}}\left(s_{i},a_{j},\cdot \right)+\epsilon T_{\text{zeros}},\text{ where }\epsilon =\frac{\gamma -\gamma_{p}}{\gamma }.$$

In Theorem 1, we will broaden the relationship between discount regularization and a weighted average transition matrix demonstrated here. We prove that discount regularization generates the same optimal policy as replacing the transition matrix with a weighted average matrix of a specific form.

Eq. 1 provides another way to view discounting as “partial termination” (Sutton and Barto, 2018). According to this classic interpretation, the sum of discounted rewards can be viewed as the sum of undiscounted rewards partially terminating with degree 1 minus the discount factor at each step. This can also be viewed as the agent transitioning to an absorbing state with probability 1 minus the discount factor at each step. To see this, observe that setting γ=1, Eq. 1 represents the agent terminating with probability $1-\gamma_{p}$ at each step.

#### 4.1.2 Equivalent Policies Regularizing the Horizon and Transition Matrix

**Figure 1:**
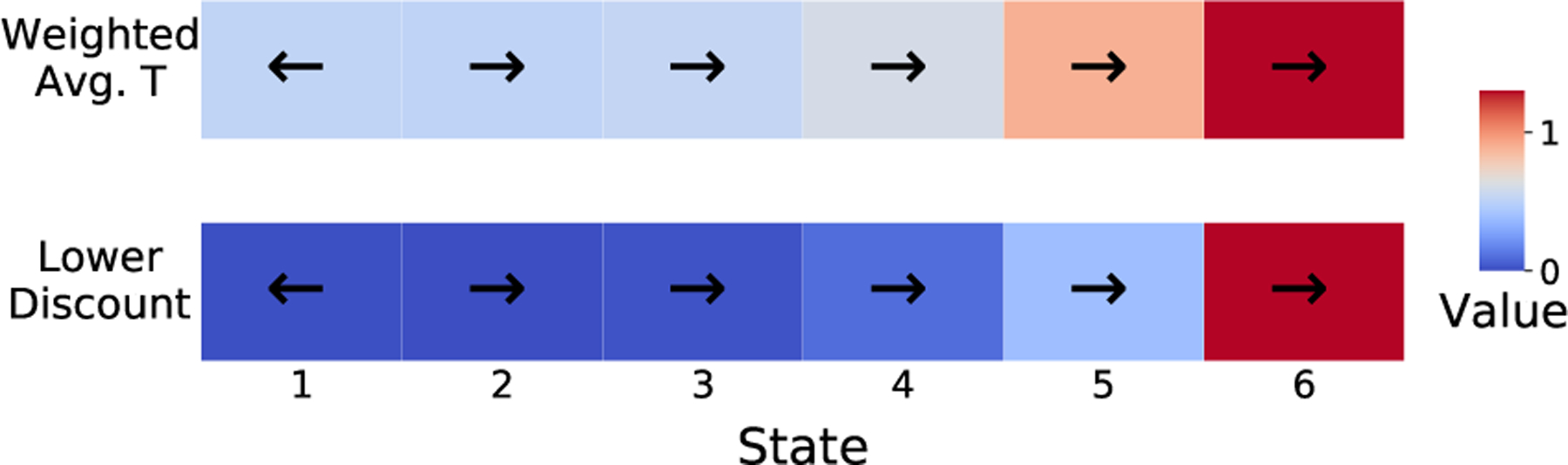
River Swim MDP described in Sec 6.1. Planning with lower discount rate or weighted average T yield different values (colors), but the same optimal policy (arrows).

The relationship between discount regularization and a weighted average transition matrix is in fact more general than discussed in the previous section. Eq. 1 shows that discount regularization is mathematically equivalent to averaging the transition matrix with a matrix of zeros, but in fact it also produces the same optimal policy as averaging the transition matrix with any regularization matrix that is the same for all states and actions when both methods use the same value of ϵ. This result is stated more precisely in Thm. 1 and illustrated in Fig. 1.

**Theorem 1** Let $M_{1}$ and $M_{2}$ be finite-state, infinite horizon MDPs with identical state space, action space, reward function. Let 0<γ<1, 0<ϵ≤1, and let $T_{reg}\left(s,a,\cdot \right)$ be any matrix used for regularization that is the same for all $\left(s,a\right)$, i.e. $T_{reg}\left(s,a,\cdot \right)=\vec{v}\;\forall \left(s,a\right)$.

If $M_{1}$ has transition function T and uses discount rate $\left(1-\epsilon \right)\gamma$ in planning and $M_{2}$ has transition function $\left(1-\epsilon \right)T+\epsilon T_{reg}$, and uses discount rate γ in planning, then $M_{1}$ and $M_{2}$ have the same optimal policy.

##### Proof

(1) *The optimal policy for all MDPs whose Bellman optimality equations differ only by added constant* c *to the reward are the same*. Consider Bellman’s optimality equation for any arbitrary state s and action a for an MDP in which constant c is added to every reward $R\left(s,a\right)$:

$$Q^{\text{*}}\left(s,a\right)=R\left(s,a\right)+c+\gamma \sum_{s^{\prime }}T\left(s,a,s^{\prime }\right)\underset{a^{\prime }}{\max}Q^{\text{*}}\left(s^{\prime },a^{\prime }\right).$$

It is known that the optimal policy of an MDP is not affected by adding the same constant c to all rewards $R\left(s,a\right)$. (See, for example, Ng et al. (1999): “constant offsets of the reward do not affect the optimal policy when γ<1”.) Proof of this step in Appendix B

It follows that the optimal policy $\pi_{\text{opt}}\left(s\right)={\text{argmax}}_{a}Q^{\text{*}}\left(s,a\right)$ is the same for all values of c. So for all values of constant c, the MDP with the Bellman optimality equation above has the same optimal policy.

(2) *The Bellman optimality equation for an MDP in which the transition matrix is regularized by taking its weighted average with a matrix* $T_{reg}$ *can be written in terms of a lower discount factor and an added constant*. Let $T_{reg}\left(s,a,\cdot \right)$ be a transition matrix that is the same for all $\left(s,a\right)$,

$$\begin{array}{l} Q^{\text{*}}\left(s,a\right)=R\left(s,a\right)+\gamma \sum_{s^{\prime }}\left[\left(\left(1-\epsilon \right)T\left(s,a,s^{\prime }\right)+\epsilon T_{reg}\left(s,a,s^{\prime }\right)\right)\underset{a^{\prime }}{\text{max}}Q^{\text{*}}\left(s^{\prime },a^{\prime }\right)\right]\\ \;=R\left(s,a\right)+\gamma \left(1-\epsilon \right)\sum_{s^{\prime }}T\left(s,a,s^{\prime }\right)\underset{a^{\prime }}{\text{max}}Q^{\text{*}}\left(s^{\prime },a^{\prime }\right)+\gamma \epsilon \sum_{s^{\prime }}T_{reg}\left(s,a,s^{\prime }\right)\underset{a^{\prime }}{\text{max}}Q^{\text{*}}\left(s^{\prime },a^{\prime }\right). \end{array}$$

Letting $c\left(s,a\right)=\gamma \epsilon \sum_{s^{\prime }}T_{reg}\left(s,a,s^{\prime }\right){\text{max}}_{a^{\prime }}Q^{\text{*}}\left(s^{\prime },a^{\prime }\right)$, Bellman’s optimality equation is:

$$Q^{\text{*}}\left(s,a\right)=R\left(s,a\right)+c\left(s,a\right)+\gamma \left(1-\epsilon \right)\sum_{s^{\prime }}T\left(s,a,s^{\prime }\right)\underset{a^{\prime }}{\text{max}}Q^{\text{*}}\left(s^{\prime },a^{\prime }\right).$$

By the assumptions of Thm. 1, $T_{reg}\left(s,a,s^{\prime }\right)$ is the same for all $\left(s,a\right)$ and is therefore a function of $s^{\prime }$ only. ${\text{max}}_{a^{\prime }}Q^{\text{*}}\left(s^{\prime },a^{\prime }\right)$ is also a function of $s^{\prime }$ only. Therefore $c\left(s,a\right)$ is a number, which we can call c,

$$c=\gamma \epsilon \sum_{s^{\prime }}\underset{\text{func. of s' only}}{\underset{︸}{T_{reg}\left(s,a,s^{\prime }\right)}}\underset{\text{func. of s' only}}{\underset{︸}{\underset{a^{\prime }}{\text{max}}Q^{\text{*}}\left(s^{\prime },a^{\prime }\right)}}=\text{constant.}$$

(3) *Setting constant c to 0 does not change the optimal policy of the resulting MDP*. By (1), replacing c with 0, the resulting new MDP with Bellman optimality equation

$$Q^{\text{*}}\left(s,a\right)=R\left(s,a\right)+\gamma \left(1-\epsilon \right)\sum_{s^{\prime }}T\left(s,a,s^{\prime }\right)\underset{a^{\prime }}{\text{max}}Q^{\text{*}}\left(s^{\prime },a^{\prime }\right)$$

has the same optimal policy as the MDP whose Bellman optimality equation has constant c added to the reward.

(4) *The resulting Bellman equation is that of an MDP with the original unregularized transition matrix* $T\left(s,a,s^{\prime }\right)$ *and reduced discount factor* $\left(1-\epsilon \right)\gamma$. Therefore, the MDP with discount rate γ and transition matrix $\left(1-\epsilon \right)T\left(s,a,s^{\prime }\right)+\epsilon T_{reg}\left(s,a,s^{\prime }\right)$ and the MDP with discount rate $\gamma \left(1-\epsilon \right)$ and transition matrix $T\left(s,a,s^{\prime }\right)$ have identical optimal policies. ■

Thm. 1 provides a deeper understanding of how discount regularization functions. At maximum regularization, $\gamma_{p}=0$ or equivalently ϵ=1, it unites two views of the relationship between bandit and MDP algorithms. An MDP algorithm with γ=0 creates a (non-adversarial) contextual bandit algorithm (Agarwal et al., 2019). Alternatively, when “the transition probability is identical... for all states and actions” in an MDP algorithm, it also forms a contextual bandit algorithm (Zanette and Brunskill, 2018). Our proof extends this equivalence beyond the bandit setting to all amounts of regularization.

Thm. 1 also reveals the limitations of discount regularization. First, the regularization matrix is the same for all state-action pairs, so it will be biased in environments where the distribution over next state varies greatly across state-action pairs. Furthermore, as we demonstrate in Sec. 4.2, this theorem leads to the result that discount regularization provides stronger regularization on state-action pairs with more data.

### 4.2 Discount Regularization Implies a Dirichlet Prior on the Transition Function

As discussed in Sec. 2, a Dirichlet prior on the transition matrix T functions as a flexible form of regularization. Given a prior on T for state-action pair $\left(s_{i},a_{j}\right)$, $T_{\text{prior}}\left(s_{i},a_{j},\cdot \right)∼\text{Dirichlet}\left(\alpha_{i,j,1},\ldots ,\alpha_{i,j,N_{s}}\right)$, the posterior mean represents a regularized form. Though simple, this generates several important insights that deepen our understanding and facilitate better regularization.

#### 4.2.1 Posterior Mean as a Weighted Average

Let $\langle c_{i,j,1},\ldots ,c_{i,j,N_{s}}\rangle$ be the transition count data observed from state $s_{i}$ to states 1 through $N_{s}$ under action $a_{j}$. Then the posterior mean of the transition matrix, ${\hat{T}}_{\begin{array}{c} \text{post}\\ \text{mean} \end{array}}$, is equal to a weighted average of the MLE transition matrix and the mean of the prior:^2^

(2) $$\begin{array}{l} {\hat{T}}_{\begin{array}{c} \text{post}\\ \text{mean} \end{array}}\left(s_{i},a_{j},\cdot \right)=\left(1-\epsilon_{i,j}\right){\hat{T}}_{MLE}\left(s_{i},a_{j},\cdot \right)+\epsilon_{i,j}T_{\begin{array}{c} \text{prior}\\ \text{mean} \end{array}}\left(s_{i},a_{j},\cdot \right),\\ \;\epsilon_{i,j}=\frac{\sum_{k=1}^{N_{s}}\alpha_{i,j,k}}{\sum_{k=1}^{N_{s}}c_{i,j,k}+\sum_{k=1}^{N_{s}}\alpha_{i,j,k}}. \end{array}$$

#### 4.2.2 Deriving the Prior Magnitude

In Thm. 1, we proved that discount regularization produces the same optimal policy as averaging the transition matrix with any regularization matrix that is the same for all states and actions. We also know from Eq. 2 that a weighted average transition matrix can be written in terms of the MLE transition matrix and a Dirichlet prior. In this section, we combine these two relationships to show that using state-action visitation rates from the data allows us to produce an empirical Bayes prior on $T\left(s,a,\cdot \right)$ that results in the same optimal policy as discount regularization.

Adapting the form of Thm. 1 to the setting where T is estimated as the MLE of the transition data, discount regularization with planning discount factor $\gamma_{p}<\gamma$ produces the same optimal policy as replacing ${\hat{T}}_{MLE}\left(s,a,\cdot \right)$ with $\left(1-\epsilon \right){\hat{T}}_{MLE}+\epsilon T_{reg}$, where $\epsilon =\frac{\gamma -\gamma_{p}}{\gamma }$ and $T_{reg}$ is the same for all $\left(s,a\right)$. Using Eq. 2, we can view this weighted average transition matrix as a posterior mean. Viewing $\left(1-\epsilon \right){\hat{T}}_{MLE}+\epsilon T_{reg}$ as a Bayesian posterior mean, the weight $\epsilon =\frac{\gamma -\gamma_{p}}{\gamma }$ equates to $\epsilon_{i,j}$ in Eq. 2.

Since discount regularization employs the same planning discount rate and consequently the same value of ϵ for every state-action pair, the prior that produces an equivalent policy also has the same value of ϵ at every state-action pair. Setting the formulas for ϵ from Eq. 1 and Eq. 2 equal and solving for the sum of the prior magnitude $\sum_{k=1}^{N_{s}}\alpha_{i,j,k}$ reveals the relationship between the planning discount factor $\gamma_{p}$ and the prior magnitude. We see that a lower planning discount factor implies a prior whose magnitude depends on the number of transitions from $\left(s_{i},a_{j}\right)$ in the data,^3^

(3) $$\sum_{k=1}^{N_{s}}\alpha_{i,j,k}=\left(\frac{\gamma -\gamma_{p}}{\gamma_{p}}\right)\sum_{k=1}^{N_{s}}c_{i,j,k}.$$

In the case of a uniform prior, which we use in our simulations, the magnitude simplifies to

$$\alpha_{i,j,k}=\left(\frac{\gamma -\gamma_{p}}{\gamma_{p}}\right)\frac{\sum_{k=1}^{N_{s}}c_{i,j,k}}{N_{s}}\;\forall k.$$

**Figure 2:**
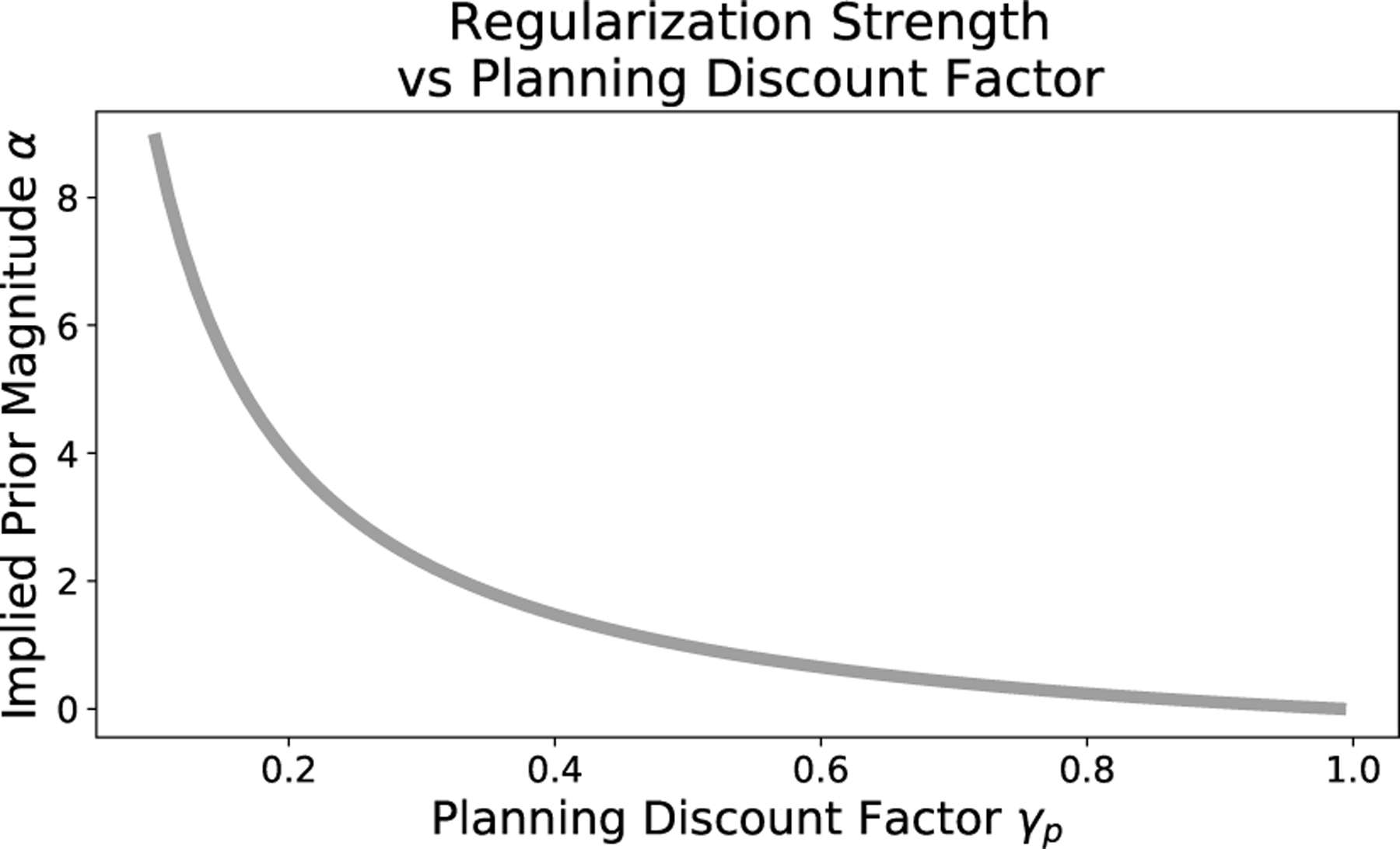
Smaller planning discount factor $\gamma_{p}$ implies larger magnitude of a uniform Dirichlet prior for an MDP with 10 states, 20 transition observations per state, and γ=0.99.

The relationship between uniform prior magnitude $\alpha_{i,j,k}$ and planning discount factor $\gamma_{p}$ for an individual state-action pair is illustrated in Fig. 2 where a smaller planning discount factor $\gamma_{p}$ implies a larger uniform prior magnitude α. Furthermore, Eq. 3 shows us that, for any planning discount factor $\gamma_{p}$, the magnitude of the corresponding Dirichlet prior is higher for state-action pairs with more data. In other words, those $\left(s,a\right)$ with more observations in the data are regularized more. Especially for data sets with uneven distribution of transition data, it may be better to use a more flexible regularization method. In Sec. 5.1, we introduce state-action-specific regularization to mitigate this issue. Note that the special case of $\gamma_{p}=0$, a type of contextual bandit, presents an exception as the implied priors for all $\left(s,a\right)$ are of infinite magnitude. This case is fundamentally different as the future is not just discounted but rather completely ignored.

Next we demonstrate that the same equivalence relationship from Thm. 1 holds in the model-free setting. We will see that by changing the setting from model-based to model-free, discount regularization can be viewed as a modified Q-function update rule instead of a prior on the transition matrix.

### 4.3 Discount Regularization as a Modified Q-Function

The equivalence between regularizing the horizon and regularizing the transition matrix arises when viewed in the model-based setting, however, this relationship extends to model-free algorithms as well. Restated in the model-free context, discount regularization functions like an added penalty or bonus on the Q-function.

**Theorem 2** Let $M_{1}$ and $M_{2}$ be finite-state, infinite horizon MDPs with identical state space, action space, and reward function. Let discount factor 0≤γ<1, regularization parameter 0<ϵ≤1, and let $Q_{reg}\left(s,a\right)$ be a function used to regularize the Q-function that is constant in $\left(s,a\right)$, i.e. $Q_{reg}\left(s,a\right)=Q_{reg}$.

If $M_{1}$ uses discount rate $\gamma_{1}=\left(1-\epsilon \right)\gamma$ and state-action value function

$$Q^{\text{*}}\left(s,a\right)=R\left(s,a\right)+\gamma_{1}E_{s^{\prime }∼T\left(S,A,\cdot \right)}\left[\underset{a^{\prime }}{\text{max}}Q^{\text{*}}\left(s^{\prime },a^{\prime }\right)|S=s,A=a\right]$$

in planning, and $M_{2}$ uses discount rate γ and state-action value function

(4) $$Q^{\text{*}}\left(s,a\right)=R\left(s,a\right)+\gamma E_{s^{\prime }∼T\left(S,A,\cdot \right)}\left[\underset{a^{\prime }}{\text{max}}\left[\left(1-\epsilon \right)Q^{\text{*}}\left(s^{\prime },a^{\prime }\right)+\epsilon Q_{reg}\left(s,a\right)\right]|S=s,A=a\right]$$

in planning, then $M_{1}$ and $M_{2}$ have the same optimal policy.

#### Proof

(1) $Q_{reg}\left(s,a\right)$ does not depend on T, so Eq. 4 is equal to:

$$Q^{\text{*}}\left(s,a\right)=R\left(s,a\right)+\gamma \epsilon Q_{reg}\left(s,a\right)+\gamma E_{s^{\prime }∼T\left(S,A,\cdot \right)}\left[\underset{a^{\prime }}{\text{max}}\left(1-\epsilon \right)Q^{\text{*}}\left(s^{\prime },a^{\prime }\right)|S=s,A=a\right].$$

(2) $Q_{reg}\left(s,a\right)$ is constant by construction, therefore $c=\gamma \epsilon Q_{reg}\left(s,a\right)=\gamma \epsilon Q_{reg}$ is constant,

$$Q^{\text{*}}\left(s,a\right)=R\left(s,a\right)+c+\gamma E_{s^{\prime }∼T\left(S,A,\cdot \right)}\left[\underset{a^{\prime }}{\text{max}}\left(1-\epsilon \right)Q^{\text{*}}\left(s^{\prime },a^{\prime }\right)|S=s,A=a\right].$$

(3) By step (1) of the proof of Thm. 1, setting c=0 does not change the optimal policy. Therefore the MDP with the following Q-function has the same optimal policy as $M_{2}$,

$$\begin{array}{l} Q^{\text{*}}\left(s,a\right)=R\left(s,a\right)+\gamma E_{s^{\prime }∼T\left(S,A,\cdot \right)}\left[\underset{a^{\prime }}{\text{max}}\left(1-\epsilon \right)Q^{\text{*}}\left(s^{\prime },a^{\prime }\right)|S=s,A=a\right]\\ \;=R\left(s,a\right)+\gamma \left(1-\epsilon \right)E_{s^{\prime }∼T\left(S,A,\cdot \right)}\left[\underset{a^{\prime }}{\text{max}}Q^{\text{*}}\left(s^{\prime },a^{\prime }\right)|S=s,A=a\right]\\ \;=R\left(s,a\right)+\gamma_{1}E_{s^{\prime }∼T\left(S,A,\right)}\left[\underset{a^{\prime }}{\text{max}}Q^{\text{*}}\left(s^{\prime },a^{\prime }\right)|S=s,A=a\right]. \end{array}$$

This is the Q-function for MDP $M_{1}$. ■

The model-based (Thm. 1) and model-free (Thm. 2) versions of the discount regularization equivalence theorem make the same assumption: the agent plans as if it transitions according to a distribution $T_{reg}\left(s,a,s^{\prime }\right)$ that is the same for all $\left(s,a\right)$ with probability ϵ at each step. This is clear in the model-based version. In the model-free setting, transitioning according to $T_{reg}$ at each step with probability ϵ equates to averaging the expected value of next state with $Q_{reg}=E_{s^{\prime }∼T_{reg}\left(S,A,\cdot \right)}\left[{\text{max}}_{a^{\prime }}Q^{\text{*}}\left(s^{\prime },a^{\prime }\right)\right]$. Despite the same assumptions, the model-based and model-free versions lead to distinct interpretations. We showed above that when viewed in the model-based setting, discount regularization functions by restricting model complexity, or acting like a prior on the transition matrix. In the model-free setting, discount regularization relates to regularization methods that penalize the value function.

As mentioned in Sec. 2, previous works have proven equivalences between different regularization methods that place a penalty or bonus on the value function. The equivalence in Thm. 2 is comparable to Proposition 1 in Amit et al. (2020). They showed that discount regularization in TD learning methods functions equivalently to a regularization term added to the objective. The formula for the regularization term differs from ours given the different setting and assumptions, however the insights are consistent. First, they note that, “This term penalizes large value estimates and therefore encourages consistent value estimates across state-action pairs which may encourage generalization by reducing the effect of spurious approximation errors.” In our Thm. 2, the modified Q-function averages the state-action value with a constant, pushing all value estimates closer together. They also conclude that “...states that are visited less often are less regularized” because the regularization term depends on the distribution of states in the data. This is exactly what we observed from the empirical Bayes prior in the model-based setting as discussed in Sec. 4.2.

### 4.4 Discount Regularization as a Truncated Lambda Return

A final interpretation of discount regularization is as an approximation to the lambda return, which is another common form of regularization in RL.

#### Discount regularization calculates an approximate value equal to a truncated lambda return.

Unlike the λ-return, discount regularization does not give the exact return for a fixed $\gamma_{p}<\gamma$. By expanding out the terms of the λ-return, we show that the approximate return under discount regularization is equal to a λ-return with truncated k-step returns.

To see this, first, expand both sums in the definition of the λ-return.


$$R_{t}^{\lambda }=\left(1-\lambda \right)\sum_{k=1}^{\infty }\lambda^{k-1}R_{t}^{\left(k\right)}\;\;=\left(1-\lambda \right)\sum_{k=1}^{\infty }\lambda^{k-1}\left[\sum_{j=0}^{k-1}\gamma^{j}R_{t+j+1}+\gamma^{k}V^{\pi }\left(S_{t+k}\right)\right]\;\;=\left(1-\lambda \right)\lambda^{0}\left[\gamma^{0}R_{t+1}+\gamma^{1}V^{\pi }\left(S_{t+1}\right)\right]\;\;+\left(1-\lambda \right)\lambda^{1}\left[\gamma^{0}R_{t+1}+\gamma^{1}R_{t+2}+\gamma^{2}V^{\pi }\left(S_{t+2}\right)\right]\;\;+\left(1-\lambda \right)\lambda^{2}\left[\gamma^{0}R_{t+1}+\gamma^{1}R_{t+2}+\gamma^{2}R_{t+3}+\gamma^{3}V^{\pi }\left(S_{t+3}\right)\right]\;\;+\cdots$$

Truncating each k-step return after the k rewards (or equivalently setting $V^{\pi }\left(S_{t+k}\right)=0\forall k$), we recover discount regularization. To see this, group the terms for each reward together then set $V^{\pi }\left(S_{t+k}\right)=0$.

First grouping terms:

$$R_{t}^{\lambda }=\left(1-\lambda \right)\gamma^{0}\left(\sum_{k=0}^{\infty }\lambda^{k}\right)R_{t+1}+\left(1-\lambda \right)\gamma^{1}\left(\sum_{k=1}^{\infty }\lambda^{k}\right)R_{t+2}+\left(1-\lambda \right)\gamma^{2}\left(\sum_{k=2}^{\infty }\lambda^{k}\right)R_{t+3}+\cdots \;\;=\gamma^{0}\left(\lambda^{0}+\lambda^{1}+\lambda^{2}+\cdots -\lambda^{1}-\lambda^{2}-\lambda^{3}-\cdots \right)R_{t+1}\;\;+\gamma^{1}\left(\lambda^{1}+\lambda^{2}+\lambda^{3}+\cdots -\lambda^{2}-\lambda^{3}-\lambda^{4}-\cdots \right)R_{t+2}\;\;+\gamma^{2}\left(\lambda^{2}+\lambda^{3}+\lambda^{4}+\cdots -\lambda^{3}-\lambda^{4}-\lambda^{5}-\cdots \right)R_{t+3}\;\;+\cdots \;\;+\left(1-\lambda \right)\lambda^{0}\;\gamma^{1}\;V^{\pi }\left(S_{t+1}\right)+\left(1-\lambda \right)\lambda^{1}\;\gamma^{2}\;V^{\pi }\left(S_{t+2}\right)+\left(1-\lambda \right)\lambda^{2}\;\gamma^{3}\;V^{\pi }\left(S_{t+3}\right)+\cdots \;\;=(\gamma \lambda {)}^{0}R_{t+1}+(\gamma \lambda {)}^{1}R_{t+2}+(\gamma \lambda {)}^{2}R_{t+3}+\cdots +\left(1-\lambda \right)\gamma \sum_{k=0}^{\infty }{\left(\lambda \gamma \right)}^{k}V^{\pi }\left(S_{t+k+1}\right)$$

(5) $$R_{t}^{\lambda }=\sum_{k=0}^{\infty }(\gamma \lambda {)}^{k}R_{t+k+1}+\left(1-\lambda \right)\gamma \sum_{k=0}^{\infty }{\left(\lambda \gamma \right)}^{k}V^{\pi }\left(S_{t+k+1}\right)$$

Set $V^{\pi }\left(S_{t+k}\right)=0$ to get a truncated λ-return: ${\hat{R}}_{t}^{\lambda }=\sum_{k=0}^{\infty }{\left(\gamma \lambda \right)}^{k}R_{t+k+1}$. Let planning discount factor $\gamma_{p}=\gamma \lambda$. Then our truncated lambda return is equal to the return under discount regularization, ${\hat{R}}_{t}^{\lambda }=\sum_{k=0}^{\infty }\gamma_{p}^{k}R_{t+k+1}$.

#### Bias of the discount regularized value

Since $R_{t}^{\lambda }$ is equal to the true return, Eq. 5, decomposes the return into the discount regularized return and a bias term. Taking the expectation over policy π to get value, the bias of the discount regularized estimate of value is $\text{Bias}\left(V_{\begin{array}{c} \text{disc}\\ \text{reg} \end{array}}\left(s\right)\right)=-E_{\pi }\left[\left(1-\lambda \right)\gamma \sum_{k=0}^{\infty }{\left(\lambda \gamma \right)}^{k}V^{\pi }\left(S_{t+k+1}\right)|S_{t}=s\right]$. The bias is the weighted average value of states expected to be visited when following policy π starting at state s, with weight decaying over time. As expected, the discount regularized value is most biased for states from which it is expected to reach high-value states soon.

The truncated lambda return also provides another illustration of the interpretation of discounting as partial termination or transitioning to an absorbing state with probability 1 minus the discount factor at each step, as we saw in Sec. 4.1.1. Setting all state values in the bias term to 0 represents a partial termination at each step. In the undiscounted setting, γ=1 and the bias term reflects partially terminating with degree 1−λ at each time step.

## 5 State-Action-Specific Regularization

We exposed in Eq. 3 that discount regularization functions like an empirical Bayes prior with the undesirable property of stronger regularization strength on state-action pairs with more data. To address this problem, we apply the model-free and model-based equivalence theorems to motivate methods that tailor the amount of regularization to each state-action pair rather than setting a global regularization parameter.

### 5.1 Model-Based Regularization Method

To avoid the issue of mismatched regularization strengths across state-action pairs, we return to the weighted average form introduced in Sec. 4.1.1 to derive a formula for state-action-specific regularization. Using this form, we calculate the MSE of the estimated transition matrix and identify the value of regularization parameter ϵ that minimizes this error separately for each $\left(s,a\right)$. While we recognize that a low-MSE transition matrix estimate does not guarantee a good policy (since some errors in T affect the optimal policy more than others),^4^ it is a reasonable step towards that goal.

We derive a closed-form expression for the MSE for the case of a uniform Dirichlet prior. We take $\text{MSE}\left(\hat{T}\left(s,a,\cdot \right)\right)$ to be the sum of the MSE of the individual elements. The derivation using the bias-variance decomposition of MSE is provided in Appendix C and the resulting form is below. Letting ${\hat{T}}_{\text{unif}}$ be the posterior mean of T under a uniform Dirichlet prior,

(6) $$\begin{array}{c} \text{MSE}\left[{\hat{T}}_{\text{unif}}\left(s_{i},a_{j},\cdot \right)\right]=\sum_{k=1}^{N_{s}}\underset{variance}{\underset{︸}{{\left(1-\epsilon_{i,j}\right)}^{2}\frac{1}{\sum_{k=1}^{N_{s}}c_{i,j,k}}T\left(s_{i},a_{j},s_{k}\right)\left(1-T\left(s_{i},a_{j},s_{k}\right)\right)}}\\ +\underset{bias^{2}}{\underset{︸}{\epsilon_{i,j}^{2}{\left(\frac{1}{N_{s}}-T\left(s_{i},a_{j},s_{k}\right)\right)}^{2}}}. \end{array}$$

Let $\epsilon_{i,j}^{\text{*}}$ be the value of the regularization parameter $\epsilon_{i,j}$ that minimizes the MSE equation. Then,

(7) $$\epsilon_{i,j}^{\text{*}}=\frac{K\left(s_{i},a_{j}\right)}{K\left(s_{i},a_{j}\right)+\sum_{k=1}^{N_{s}}c_{i,j,k}},\text{ where }K\left(s_{i},a_{j}\right)=\frac{\sum_{k=1}^{N_{s}}T\left(s_{i},a_{j},s_{k}\right)\left(1-T\left(s_{i},a_{j},s_{k}\right)\right)}{\sum_{k=1}^{N_{s}}{\left(\frac{1}{N_{s}}-T\left(s_{i},a_{j},s_{k}\right)\right)}^{2}}.$$

The first term of Eq. 6 is the contribution of the MLE's variance to the error, in this case the only source of variance. The second term is the bias introduced by regularization. The strength of regularization $\epsilon_{i,j}$ controls the trade-off between the bias and variance. The variance is driven by the amount of data $\sum_{k=1}^{N_{s}}c_{i,j,k}$ both through its role in setting the amount of regularization and as a factor inversely impacting the variance term. Both bias and variance are impacted by the true transition distribution $T\left(s_{i},a_{j},\cdot \right)$. A deterministic $T\left(s_{i},a_{j},\cdot \right)$ maximizes bias for a given $\epsilon_{i,j}$, but results in $\epsilon_{i,j}^{\text{*}}=0$ (since $T\left(s_{i},a_{j},s_{k}^{'}\right)$ $\left(1-T\left(s_{i},a_{j},s_{k}^{'}\right)\right)=0$ for all $s_{k}^{'}$). At the other extreme, if $T\left(s_{i},a_{j},\cdot \right)$ is uniform, variance is maximized for a given $\epsilon_{i,j}$ but there is no bias, so we default to $\epsilon_{i,j}^{\text{*}}=1$. Intermediate values of $\epsilon_{i,j}^{\text{*}}$ trade off between bias and variance. A uniform prior on $T\left(s_{i},a_{j},\cdot \right)$ with state-action-specific parameter $\epsilon_{i,j}^{\text{*}}$ improves upon discount regularization by setting the parameters locally for each state-action pair rather than forcing one global regularization parameter.

Furthermore, there is no parameter tuning required, simply a plugin estimate for T (e.g. the MLE). In practice, the true transition matrix T is not known and must be estimated. We may worry that in the low data regimes in which regularization is required, the estimate of T will not be good enough to estimate $\epsilon_{i,j}^{\text{*}}$. Nonetheless, our empirical examples in Sec. 6.3 demonstrate that our formula for $\epsilon_{i,j}^{\text{*}}$ leads to a reduction in loss over a single global regularization parameter.

Note that the state-action-specific parameter $\epsilon_{i,j}^{\text{*}}$ combined with regularization matrix $T_{reg}$ does not map directly to a state-action-specific discount factor. Step (2) of the proof of Thm. 1 depends on $c=\gamma \epsilon \sum_{s^{\prime }}T_{reg}\left(s,a,s^{\prime }\right){\text{max}}_{a^{\prime }}Q^{\text{*}}\left(s^{\prime },a^{\prime }\right)$ being constant. Otherwise, we cannot set c=0 and expect the resulting MDP (which represents discount regularization) to have the same optimal policy. A state-action-specific discount factor breaks this equivalence.

### 5.2 Model-Free Regularization Methods

In the model-free setting, as in the model-based setting, a fixed universal regularization parameter like the on used in discount regularization can cause a mismatch in regularization strengths when the count data is uneven across state-action pairs. To address this problem, we set regularization strength separately for each $\left(s_{i},a_{j}\right)$ based on the Q-function and amount of data. A natural choice for state-action specific parameter $\epsilon_{i,j}^{\text{*}}$ in the modelfree setting is the value that minimizes the mean squared error in Q separately for each state-action pair. We investigate this approach, calculating the analytical solution by two different methods.

We demonstrate the performance of these two calculations for $\epsilon_{i,j}^{\text{*}}$ on fitted Q-iteration (FQI, Ernst et al. 2005). In FQI, $Q\left(s,a\right)$ is estimated for each observation ${\left\{s,a,s^{\prime }\right\}}_{d}$ in the data set, $q_{d}=R\left(s_{d},a_{d}\right)+\gamma \;{\text{max}}_{a}\hat{Q}\left(s_{d}^{'},a\right)$.^5^ FQI alternates between using $\hat{Q}$ to calculate ${\left\{q\right\}}_{d}$ and updating $\hat{Q}$ to minimize the MSE across ${\left\{q\right\}}_{d}$. To regularize, we use the weighted average Q-function from Eq. 4 in calculating ${\left\{q\right\}}_{d}$, with $Q_{reg}$ equal to the average value across states. This is equivalent to the case of a uniform regularization matrix $T_{reg}\left(s_{i},a_{j},\cdot \right)$ in the model-based setting. We update $\epsilon_{i,j}^{\text{*}}$ at each iteration, minimizing the sum of errors in estimates of $Q\left(s_{i},a_{j}\right)$ across the data. The procedure is detailed in Algorithm 1.

#### 5.2.1 Regularization Parameter Calculation: Method 1

To derive $\epsilon_{i,j}^{\text{*}}$, we first calculate the sum of squared errors (SSE). Given the iterative procedure, we derive the SSE and resulting expression for $\epsilon_{i,j}^{\text{*}}$ for a fixed policy π and associated Q-function $Q^{\pi }\left(s_{i},a_{j}\right)$, which is updated at every step.

The SSE is the sum across all data tuples of the squared errors incurred by estimating the Q-function for tuple $\left\{s_{i},a_{j},s_{k}^{'}\right\}$ with weighted-average Q-function $R\left(s_{i},a_{j}\right)+\gamma \left(1-\epsilon_{i,j}\right)Q^{\pi }\left(s_{i},\pi \left(s_{i}\right)\right)+\gamma \epsilon_{i,j}\frac{1}{N_{s}}\sum_{k^{\prime }=1}^{N_{s}}Q^{\pi }\left(s_{k^{\prime }},\pi \left(s_{k^{\prime }}\right)\right)$. Therefore, the contribution of each state-action pair to the error calculation is weighted by how frequently it appears in the data. This results in the expression,

(8) $$\begin{array}{l} SSE=\sum_{i,j}\sum_{k=1}^{N_{s}}c_{i,j,k}\left(R\left(s_{i},a_{j}\right)+\gamma \left(1-\epsilon_{i,j}\right)Q^{\pi }\left(s_{k},\pi \left(s_{k}\right)\right)\right.\\ \;+\gamma \epsilon_{i,j}\frac{1}{N_{s}}\sum_{k^{\prime }=1}^{N_{s}}{\left.Q^{\pi }\left(s_{k^{\prime }},\pi \left(s_{k^{\prime }}\right)\right)-Q^{\pi }\left(s_{i},a_{j}\right)\right)}^{2}. \end{array}$$

$$\begin{array}{l} \bar{\underline{\mathrm{Algorithm}1\text{ FQI with State-Action-Specific Regularization}}}\\ \text{ 1: Initialize }\hat{Q}\left(s,a\right)=0\forall \left(s,a\right)\\ \text{ 2: Initialize }\epsilon^{*}(s,a)\text{ randomly}\\ \text{ 3: }\mathrm{for}\text{ i in num\_iters }\mathrm{do}\\ \text{ 4: Start with P}=\{\}\\ \text{ 5: }Step\;1:\;Estimate\;Q\;for\;each\;(s,a)\;data\;observation\\ \text{ 6: }\mathrm{for}\text{ every tuple }\left\{\text{s,a,s'}\right\}\text{ in data set }\mathrm{do}\\ \text{ 7: }q=R(s,a)+\gamma \left(1-\epsilon_{s,a}^{*}\right)\max_{a}\hat{Q}\left(s^{\prime },a\right)+\gamma \epsilon_{s,a}^{*}{\mathrm{average}}_{s^{\prime \prime }}\left(\max_{a^{\prime }}\hat{Q}\left(s^{\prime \prime },a^{\prime }\right)\right)\\ \text{ 8: Add }\left\{\left(\text{s},\text{a}\right),\text{q}\right\}\}\text{ to P}\\ \text{ 9: }\mathrm{end}\;\mathrm{for}\\ \text{10: }Step\;2:Learn\;Q\\ \text{11: Fit linear regression model with one-hot }\left(\text{s,a}\right)\text{ encoding as features and q as dependent variable}\\ \text{12: Set }\hat{Q}\left(s,a\right)\text{ equal to the coefficient for (s,a) from the regression model}\\ \text{13: }Step\;3:Update\;\epsilon_{s,a}^{*}.\text{ Set }\epsilon_{s,a}^{*}\text{ equal to the diagonal values of matrix }ℰ\text{calculated by}\\ \text{ Eq }9,\text{using }\hat{Q}\text{ from Step 2 and }\pi^{*}(s)=argmax_{a}\hat{Q}(s,a).\\ \text{14: }\mathrm{end}\;\mathrm{for}\\ \underline{\text{15: }\mathrm{return}\text{ value,optimal policy based on learned Q}} \end{array}$$

For each $\left(s_{i},a_{j}\right)$, we solve for the value $\epsilon_{i,j}^{\text{*}}$ that minimizes Eq. 8 by setting the derivative equal to zero. Then we re-write the expression in matrix form to solve for all $\epsilon_{i,j}^{\text{*}}$ simultaneously. We use the following notation for the matrix equation: ℰ is an $N_{s}N_{a}\times N_{s}N_{a}$ matrix with $\epsilon_{i,j}$ across the main diagonal, $v_{avg}^{\pi }$ is the average state value under policy π, C is the $N_{s}N_{a}\times N_{s}$ matrix of transition counts (one row for every $\left(s,a\right)$, one column for every $s^{\prime }$), Π is a matrix mapping $Q^{\pi }$ to $V^{\pi }$ for fixed policy π. We get,

(9) $$\begin{array}{l} ℰ={\left[\gamma^{2}\text{Diag}\left(C\underset{\text{elementwise square}}{\underset{︸}{{\left[{\text{Π}}^{T}Q^{\pi }\right]}_{sq}}}\right)-2\gamma^{2}v_{avg}^{\pi }Diag\left(C{\text{Π}}^{T}Q^{\pi }\right)+\gamma^{2}{\left(v_{avg}^{\pi }\right)}^{2}Diag\left(C1_{N_{s}\times 1}\right)\right]}^{-1}\\ \left[\gamma Diag\left(R\right)C{\text{Π}}^{T}Q^{\pi }+\gamma^{2}Diag\left(C\underset{\text{elementwise square}}{\underset{︸}{{\left[{\text{Π}}^{T}Q^{\pi }\right]}_{sq}}}\right)1_{N_{s}N_{a}\times 1}-\gamma Diag\left(C{\text{Π}}^{T}Q^{\pi }\right)Q^{\pi }\right.\\ \left.-\gamma v_{avg}^{\pi }Diag\left(R\right)C1_{N_{s}\times 1}-\gamma^{2}v_{avg}^{\pi }Diag\left(C{\text{Π}}^{T}Q^{\pi }\right)1_{N_{s}N_{a}\times 1}+\gamma v_{avg}^{\pi }Diag\left(C1_{N_{s}\times 1}\right)Q^{\pi }\right]. \end{array}$$

Derivations for both SSE and ℰ are in Appendix D.

#### 5.2.2 Regularization Parameter Calculation: Method 2

We also calculate the MSE of $Q^{\pi }\left(s,a\right)$ using the assumption that the transition count data follows a multinomial distribution, similar to the model-based case. For each $\left(s,a\right)$, we separately calculate the bias and variance resulting from estimating the Q-function for tuple $\left\{s_{i},a_{j},s_{k}^{'}\right\}$ with weighted-average Q-function

$$R\left(s_{i},a_{j}\right)+\gamma \left(1-\epsilon_{i,j}\right)\sum_{k=1}^{N_{s}}T\left(s_{i},a_{j},s_{k}\right)Q^{\pi }\left(s_{k},\pi \left(s_{k}\right)\right)+\gamma \epsilon_{i,j}\frac{1}{N_{s}}\sum_{k^{\prime }=1}^{N_{s}}Q^{\pi }\left(s_{k^{\prime }},\pi \left(s_{k^{\prime }}\right)\right).$$

Like in the model-based case, T is estimated from the transition counts in the data, which is assumed to be multinomially distributed. In this case, each state-action pair contributes equally to the error, rather than each data tuple contributing equally as method 1. The MSE derivation for this method is in Appendix D.3.

These methods perform similarly to each other in our simulations, but do not perform as well as the model-based approach or even a fixed global regularization parameter. We discuss the reasons in Sec. 6.4.3.

#### 5.2.3 Connections to Pessimism

By setting regularization strength inversely proportional to the transition count in the data, our method resembles pessimism however, it does not satisfy the definitions of point-wise or Bellman-consistent pessimism in Jin et al. (2021) and Xie et al. (2021), respectively.

##### Point-wise Pessimism.

In the pessimistic value iteration meta-algorithm introduced in Jin et al. (2021), pessimism is encoded as a state-action-specific penalty applied to the Bellman operator at each step of value iteration. Specifically, this penalty is instantiated by subtracting a ξ-uncertainty quantifier $\text{Γ}\left(s,a\right)$, a function that bounds the absolute value of the difference between the true Bellman operator and its estimate using the data with probability 1−ξ.^6^

The Bellman operator ${\hat{B}}_{\epsilon^{*}}\hat{V}\left(s,a\right)$ in our method can be framed similarly. In the model-free setting, we have:

$${\hat{B}}_{\epsilon^{*}}\hat{V}\left(s,a\right)=R\left(s,a\right)+\gamma \underset{a^{\prime }}{\text{max}}\left(\left(1-\epsilon_{s,a}^{\text{*}}\right)\hat{Q}\left(s^{\prime },a^{\prime }\right)+\epsilon_{s,a}^{\text{*}}Q_{reg}\right)\;\;=\underset{\hat{B}\hat{V}\left(s,a\right)}{\underset{︸}{R\left(s,a\right)+\gamma \underset{a^{\prime }}{\text{max}}\hat{Q}\left(s^{\prime },a^{\prime }\right)}}+\underset{\text{penalty/bonus}}{\underset{︸}{\gamma \epsilon_{s,a}^{\text{*}}\left(Q_{reg}-\underset{a^{\prime }}{\text{max}}\hat{Q}\left(s^{\prime },a^{\prime }\right)\right)}}$$

We must have $\text{Γ}\left(s,a\right)\geq 0$ in order to be an uncertainty quantifier because it upper bounds an absolute value. This would equate to a negative or zero “penalty/bonus” term in the equation above. Our penalty term, however, can be either positive or negative, pushing estimated Q-values towards $Q_{reg}$ rather than strictly decreasing them based on uncertainty. Thus it does not fit the definition and our method is not pointwise pessimistic. Furthermore, because the penalty term in our method is not a ξ-uncertainty quantifier, we cannot apply the sub-optimality guarantee in Theorem 4.2 of Jin et al. (2021).

The performance of pessimistic value iteration depends on finding an uncertainty quantifier that tightly bounds the error in the Bellman operator, but in practice this may be difficult to find. While our method does not come with performance guarantees, it provides a methodology for how to compute a penalty or bonus for the value equation. We do note, however, that a weakness of our method is that it requires a plugin or bootstrapping to estimate T, which is required to compute the value of $\epsilon_{s,a}^{\text{*}}$.

##### Bellman-consistent Pessimism.

Our algorithm is also not pessimistic under the weaker assumptions of Bellman-consistent pessimism. In this approach from Xie et al. (2021), the value of the starting state under any policy is estimated using the most pessimistic function in the space of value functions with low Bellman error. Then the policy that maximizes this pessimistic value estimate of the starting state is chosen. In our method, it is not required that the choice of $Q_{reg}$ and the resulting value of $\epsilon^{\text{*}}$ that minimizes the MSE at the initial state be pessimistic.

Bellman-consistent pessimism applies to arbitrary function approximation and is well-suited for complex, higher-data settings, while our method provides simple approach appropriate for the low-data settings where discount regularization is generally beneficial. The main theoretical guarantee in Xie et al. (2021) requires searching over the policy space as well as evaluating each policy over all functions in the value function class with low Bellman error, which is not practical to implement. The version of the algorithm adapted for practical implementation avoids searching over the policy space, but still requires “access to a (regularized) loss minimization oracle over the value function class.” They demonstrate that this can be efficiently implemented in linear function approximation, making Bellman-consistent pessimism a reasonable choice for settings where linear function approximation is used. Our method is more appropriate for small-data settings where function approximation is not appropriate, albeit without theoretical guarantees on the resulting performance.

## 6 Simulations

In this section, we empirically confirm our equivalence theorems and demonstrate the performance of our regularization methods in an offline setting.

### 6.1 Tabular Environments

We demonstrate our results on three common environments from the RL literature. The first comes from the initial work proposing discount regularization. We choose this environment to demonstrate the limitations of discount regularization even in an environment where it is known to be beneficial. We choose the other two because of their differences in structure, connectivity, and rewards to ensure that our results hold in diverse environments.

#### 10-State Random Chain.

The first environment is a distribution over MDPs and we sample one before generating each data set in the examples that follow. Jiang et al. (2015) empirically demonstrated the benefits of discount regularization on this randomly generated 10-state, 2-action MDP. For each state-action pair, 5 successor states are chosen at random to have nonzero transition probability. These probabilities are drawn independently from Uniform[0,1] and normalized to sum to one. The rewards are sampled independently from Uniform[0,1].

#### River Swim.

**Figure 3:**
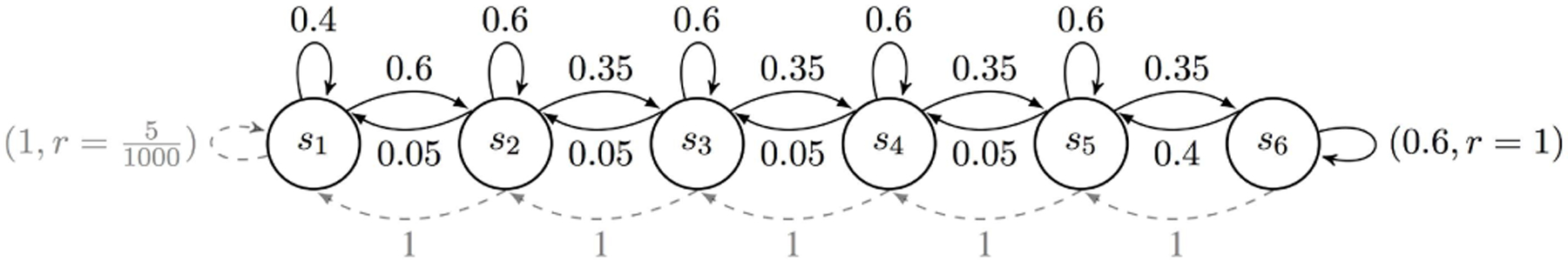
River Swim. Image from Osband et al. (2013).

This common tabular environment described in Osband et al. (2013) consists of six states and two actions, as illustrated in Figure 3. The agent can attempt to swim right “against the current” towards the larger reward, or swim left with probability 1 towards the smaller reward.

#### GridWorld.

The “GridWorld” environment is a modification of the environment with the same name from Amit et al. (2020). Like the 10-State Random Chain, it is a distribution over MDPs. The state space is a 4x4 grid where the agent’s actions are left, right, up, or down. To construct the MDP, a probability $p_{s}$ is drawn uniformly at random from [0,1] for each state s. With probability $p_{s}$, the agent moves in the desired direction if possible and otherwise remains in the same state. With probability $1-p_{s}$, the agent transitions to a successor state that is randomly chosen for each state when the MDP is constructed. A single high-reward state is chosen at random to have a reward of 1, while all other states have rewards drawn uniformly at random from [−0.5,0.50].

### 6.2 Cancer Simulator Environment

We confirm our analysis in a larger, more realistic setting, using a cancer simulator developed by Ribba et al. (2012), as implemented by Gottesman et al. (2020). The simulator is based on data from patients with a type of tumor called low-grade gliomas (LGG). We use the version for chemotherapy drug TMZ. The structure of the model is based on 21 patients and parameters for the TMZ version are fit using data from 24 patients, with the remaining 96 held out for validation.

The state space consists of four dimensions: measurements for three different tumor tissue types and the drug concentration. We discretize the states by dividing each dimension into quartiles. The two actions represent whether or not to administer the chemotherapy drug TMZ at each time step, which represents one month. The reward at each time step is the reduction in total tumor size from the previous time step, minus a penalty for administering treatment at that time step. In the batch data, treatment at each time step is determined by a draw from the binomial distribution with treatment probability p. We compare regularization methods across a range of parameter choices: amount of stochasticity in the transition between states, magnitude of penalty to the reward for administering chemotherapy, noise in the starting state, and probability p of treatment in the batch data.

### 6.3 Model-Based Method Simulations

We showed analytically that planning under a reduced discount factor functions as a prior on the transition matrix with higher magnitude for state-action pairs with more transition observations. We then proposed a better way to regularize by deriving an explicit formula for a uniform prior that minimizes that transition matrix MSE locally for each state-action pair. Next we confirm our results empirically.

First we demonstrate that the equality in Thm. 1 holds. We then compare the performance of (1) discount regularization, (2) a uniform prior on T with equal magnitude for all state-action pairs, and (3) our state-action-specific regularization on the three simple tabular examples and the medical cancer simulator.

#### 6.3.1 Procedure

To assess performance in each environment, we follow the procedure in Jiang et al. (2015). We repeatedly sample data sets from the true MDP. (A new MDP is sampled every time in the case of the 10-State Random Chain and GridWorld.) For each, we estimate the transition matrix from the data and assume the reward function is known. Then for a range of regularization strengths (ϵ or γ) we regularize the transition matrix separately using (1) discount regularization or (2) a uniform prior with constant magnitude across state-action pairs. We also regularize by (3) a uniform prior with state-action specific parameter. We then calculate the optimal policy. We compute the loss by taking the difference between the value of the true optimal policy in the true MDP and the value of the policy found in the estimated, regularized MDP, evaluated in the true MDP, and then averaging across states. The state-action-specific uniform prior is not dependent on a regularization parameter so we plot the single average loss value horizontally.

#### 6.3.2 Empirical Demonstration of Equivalence Theorem: Discount Regularization and Uniform Prior on Transition Matrix Yield Identical Optimal Policies

**Figure 4:**
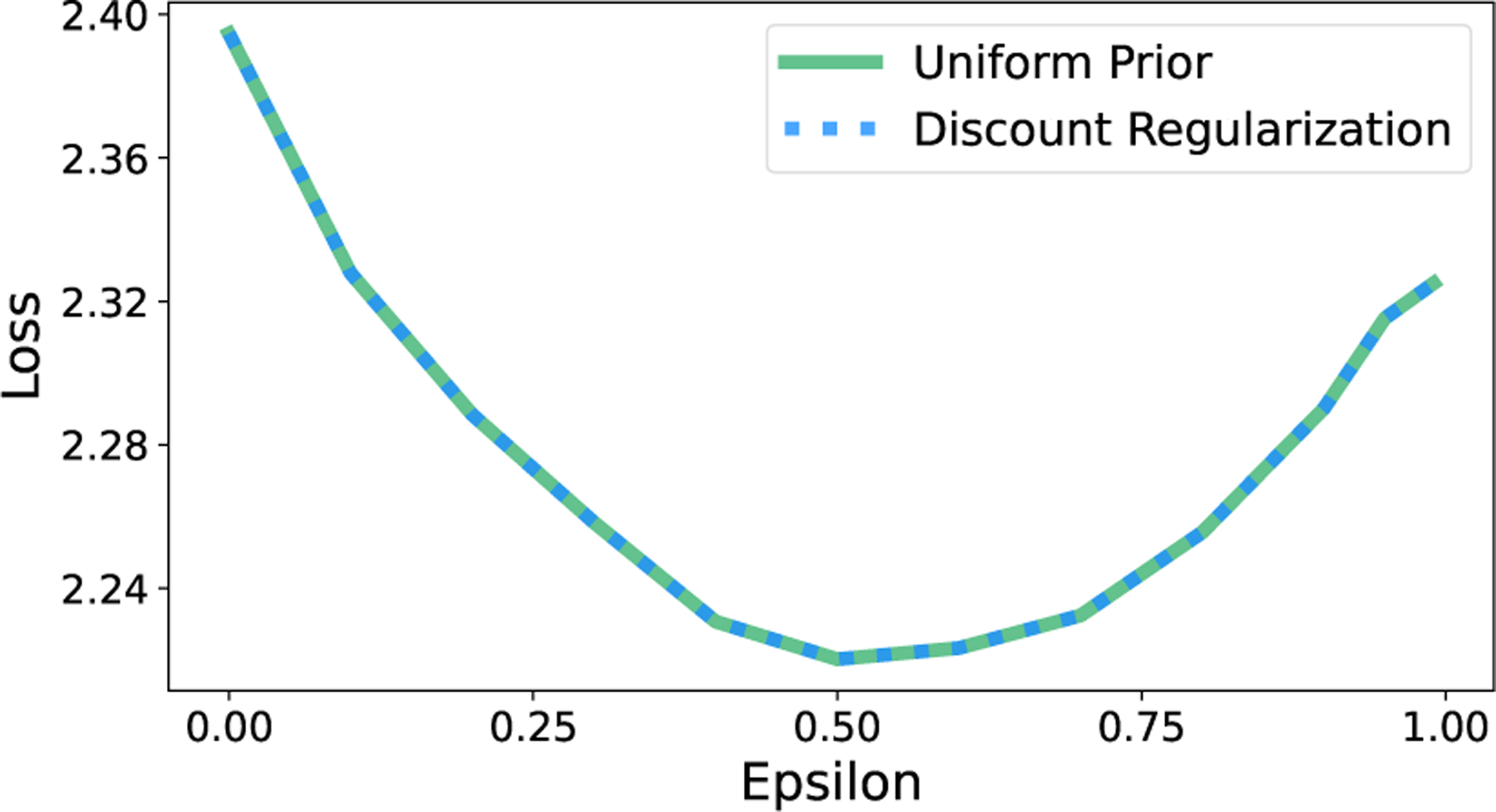
Discount regularization and a uniform prior on the transition matrix yield identical policies in model-based RL when transition count data are equal for all state-action pairs.

First, we empirically confirm our result from Thm. 1. When the implied value of ϵ is the same for all state-action pairs, a uniform prior on T yields the same optimal policy as a planning discount factor of $\gamma \left(1-\epsilon \right)$. As per Eq. 3, we enforce equal ϵ across state-action pairs by sampling data sets with equal numbers of transition observations across state-action pairs. As demonstrated for the 10-State Random Chain environment in Fig. 4, loss is identical for both methods, as is expected for identical policies.

In the examples that follow, we relax the requirement of equal data across state-action pairs to compare methods under a more realistic data distribution.

#### 6.3.3 Results: Problems with Discount Regularization

##### Discount Regularization performs poorly on data sets with uneven coverage across state-action pairs.

**Figure 5:**
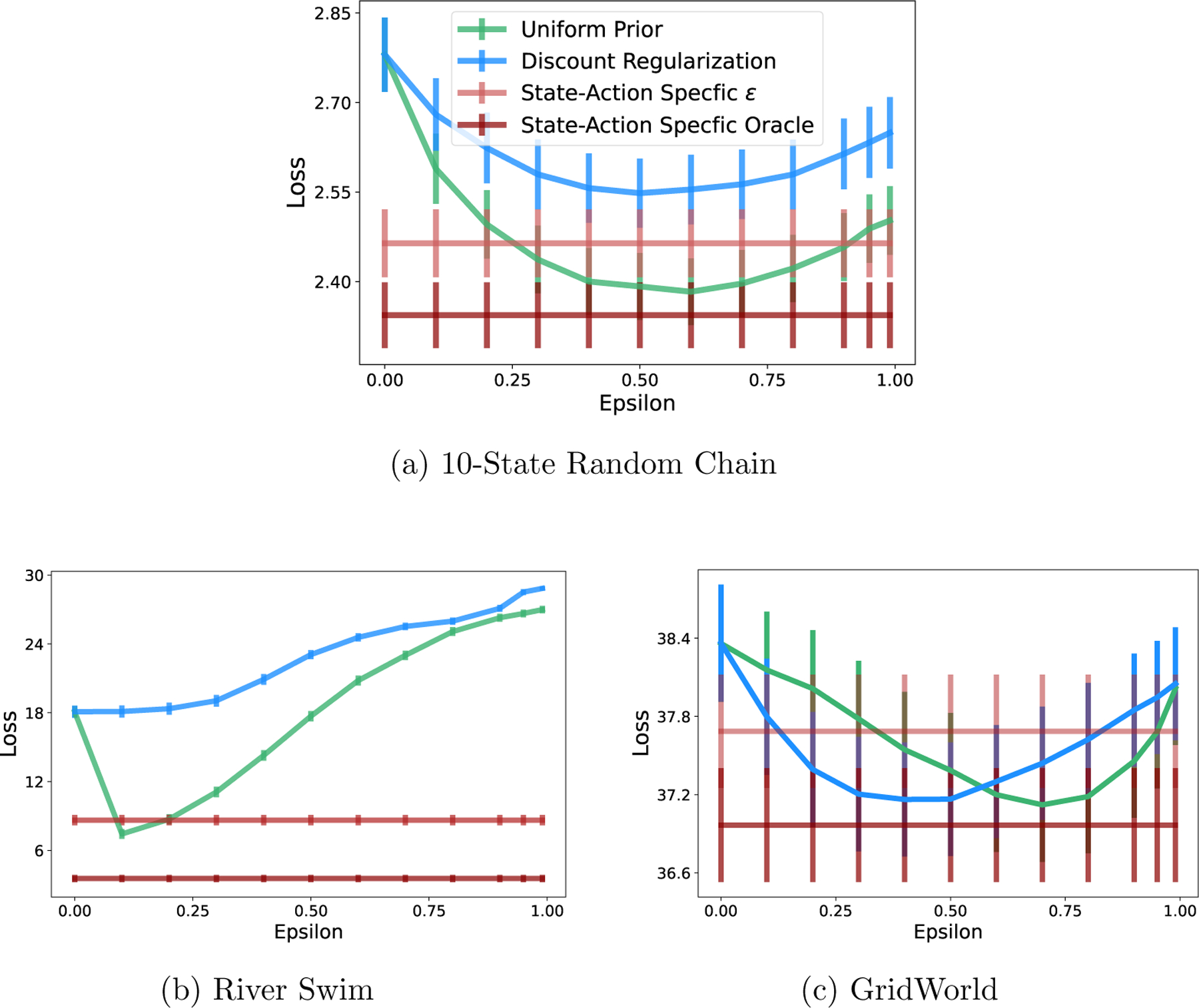
A uniform prior on the transition matrix performs equally to or better than discount regularization in all three environments. A state-action-specific uniform prior performs close to or better than a uniform prior with global regularization parameter ϵ. “Oracle” represents state-action specific $\epsilon^{\text{*}}$ calculated using the true T. Calculated for 5,000 data sets.

In real-world conditions, it is unlikely that a data set will have equal numbers of transition observations across state-action pairs. In this case, recall that discount regularization functions as a prior with higher magnitude for state-action pairs with more data (Eq. 3). We compare this with a uniform prior on the transition matrix with equal magnitude for all state-action pairs. Fig. 5 shows the loss for each method across a range of values of ϵ (regularization strengths) for the three tabular environments. In these examples, the transition data is generated with starting state and action chosen uniformly at random, but transition counts are not enforced to be equal across state-action pairs. Even with transition data that is not heavily skewed away from uniform, the uniform prior with fixed magnitude generates policies that generally perform better (lower loss) in the true environment across a range of regularization strengths. Loss is significantly lower for a uniform prior compared to discount regularization in first two environments and similar in the case of the GridWorld environment.

##### Discount regularization performs poorly when the transition distribution differs greatly across states and/or actions.

**Figure 6:**
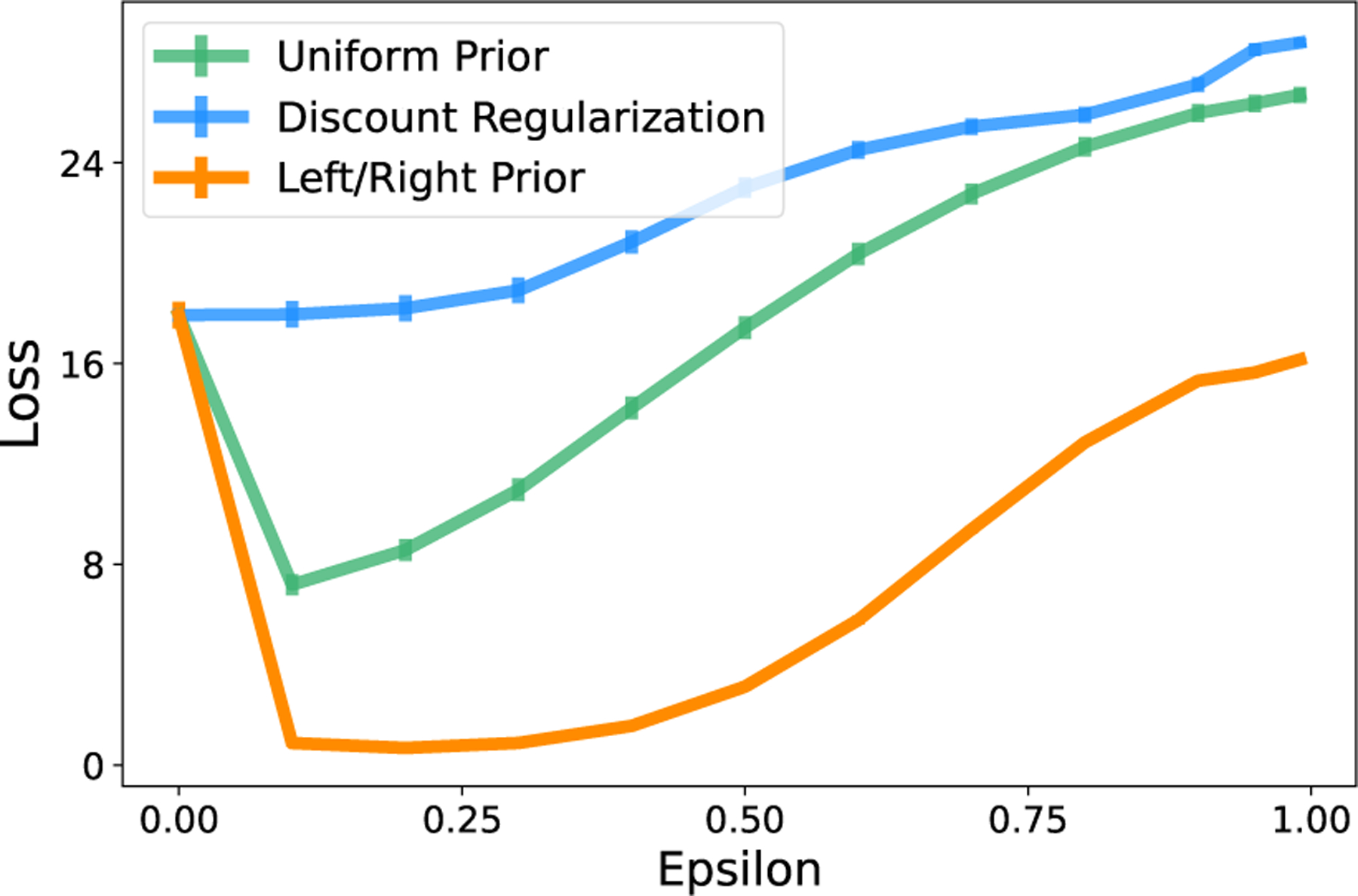
River Swim Environment. When we have knowledge about the environment, a prior chosen based on expert knowledge (the ‘Left/Right Prior’) can perform better.

In addition to poor performance in skewed data sets, discount regularization does not perform well in cases where the implied prior, which has the same distribution for all $\left(s,a\right)$, does not match the ground truth. For example, a domain expert may have knowledge that some state transitions are likely or others are impossible. Consider the case of River Swim. If a domain expert knows that Action 1 generally causes the agent to go left and Action 2 generally causes the agent to go right, we may choose a different prior on each action. For example, consider a prior on Action 1 that deterministically moves the agent left and the prior on Action 2 that deterministically moves the agent right. Fig. 6 compares the loss for this deterministic “left/right prior” with the other methods. Unsurprisingly, this hand-chosen prior results in lower loss than the methods which assume equal transition distributions for all states and actions.

#### 6.3.4 Results: Our Method Provides Simple and Flexible State-Action-Specific Regularization

Performance depends not only on choosing an appropriate regularizer for the data set and environment, but also on setting the parameters correctly.

##### Our method avoids parameter tuning.

Minimizing the transition matrix MSE equation with respect to regularization parameter $\epsilon_{i,j}$ yields an explicit formula for the parameter $\epsilon_{i,j}^{\text{*}}$, Eq. 7. This expression for $\epsilon_{i,j}^{\text{*}}$ depends inversely on the number of transition observations in the data, which allows for reduction in regularization with increased data. The only quantity we lack is an estimate for T, which can be approximated by the MLE. Alternatively, we can model T from the data then sample from the posterior, choosing $\epsilon_{i,j}$ to minimize the MSE (Eq. 6) across the sampled estimates of T. Figures in this section reflect this approach. This is preferable to cross validation not only because it provides a simple, analytic form, but also because the situations in which regularization is beneficial generally involve few transition observations per state-action pair, resulting in insufficient amounts of data to divide into training and validation sets.

The gap between “state-action specific ϵ” loss and the “state-action specific oracle” loss in Fig. 5 is the difference in performance when using an estimate of T versus the true value. As expected, loss is higher when estimating T, however it still achieves loss near the minimum that can be achieved with a global regularization parameter without the risk of incurring the higher loss values that can result from an incorrectly-set global parameter.

**Figure 7:**
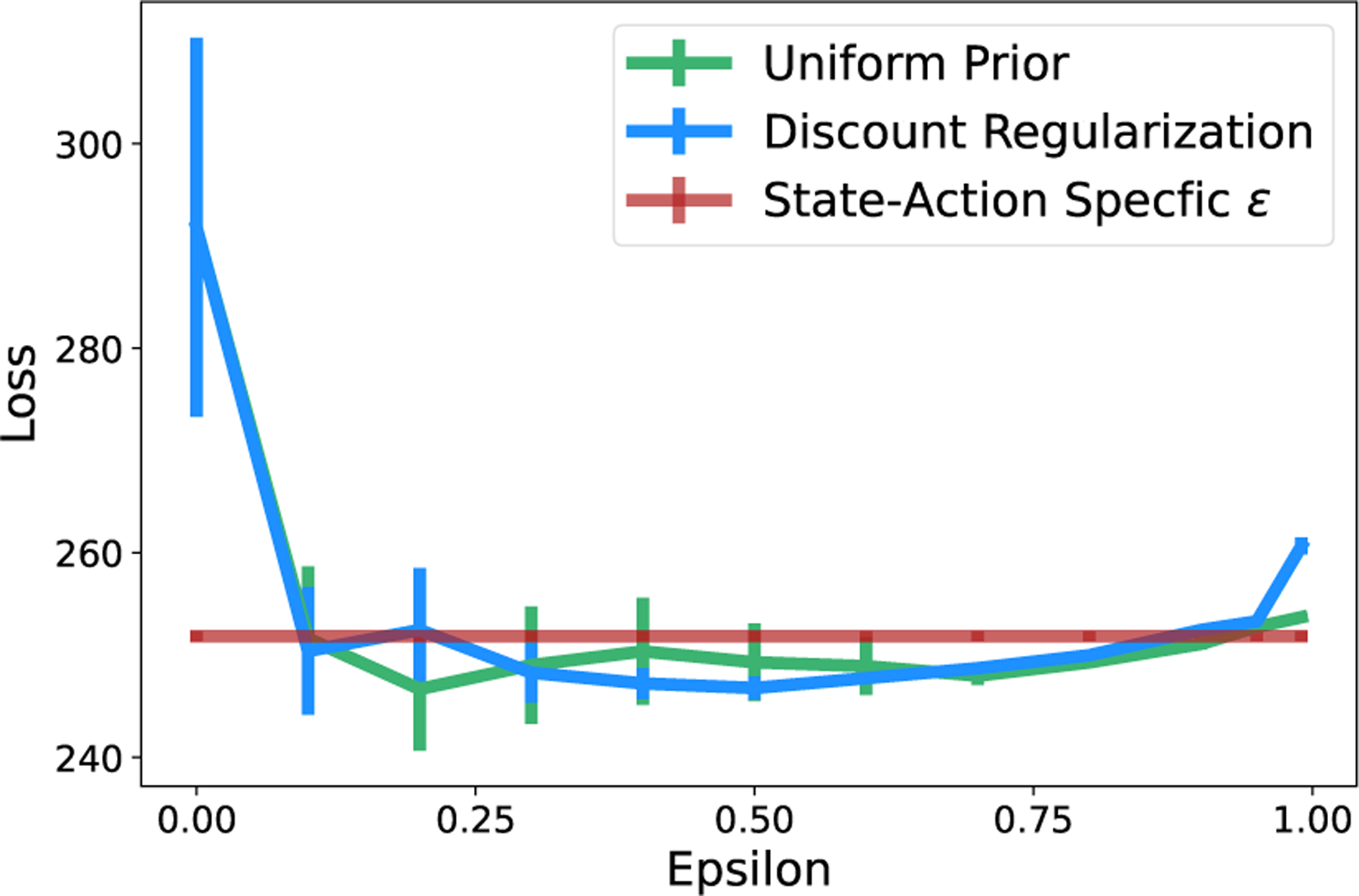
Cancer Simulator. State-action-specific regularization achieves near-minimum loss while avoiding the high loss resulting from incorrectly-set parameters.

The benefit of avoiding parameter tuning is also illustrated in the results from the cancer simulator in Fig. 7. Across variations in parameters, the two methods with global parameters performed similarly. However with both global methods, if ϵ is set incorrectly then the loss can be significantly higher. This makes state-action specific regularization particularly appealing, achieving loss near the minimum of all methods with the parameters set globally, but without tuning.

##### Our method remedies the issue of stronger regularization for state-action pairs with more data.

Because the formula for $\epsilon_{i,j}^{\text{*}}$ is state-action-specific, it allows the flexibility to adjust the regularization amount separately across state-action pairs with different amounts of data and different transition distributions. This is particularly important as most real-world data sets have uneven distributions, and enforcing equal regularization across state-action pairs in that case impedes performance.

Returning to Fig. 5, we demonstrate that our state-action-specific regularization reduces loss without parameter tuning. The horizontal line for “State-action-specific ϵ” represents the loss when regularization parameter $\epsilon_{i,j}$ is set separately for each state-action pair. A state-action-specific regularization parameter yields loss that outperforms discount regularization and is close to or outperforms a uniform prior of constant magnitude.

### 6.4 Model-Free Method Simulations

Discount regularization is used in both model-based and model-free settings, so we extend our simulations to a model-free setting. As in the model-based setting, we first demonstrate the equivalence theorem, then compare the performance of discount regularization with our state-action specific methodology. We then discuss the reasons that model-based outperforms model-free in the low-data settings that we consider.

#### 6.4.1 Procedure

We follow an analogous procedure as in the model-based case, adapted to the model-free RL method FQI. As above, we repeatedly sample data from the true MDP. For each data set, for a range of regularization strengths (ϵ or γ), we run FQI using either (1) a lower discount factor or (2) a weighted average Q-function update reflecting Eq. 4. The reward function is assumed known. Loss is the difference between the value of the true optimal policy and the value of the policy found with the regularized version of FQI, both evaluated in the true MDP, averaged across states. The state-action-specific method is not dependent on a regularization parameter so we plot the single average loss value horizontally in Fig. 9.

Note that unlike the model-based setting where any planning discount factor $\gamma_{p}$ maps to a prior on the transition matrix, there is no simple mapping between the planning discount factor and an implied prior in the model-free case. Consequently our simulations lack an equivalent comparison between discount regularization and a fixed prior of equal magnitude for all state-action pairs.

#### 6.4.2 Empirical Demonstration of Equivalence Theorem: Discount Regularization and Weighted average Q-function Yield Identical Optimal Policies

**Figure 8:**
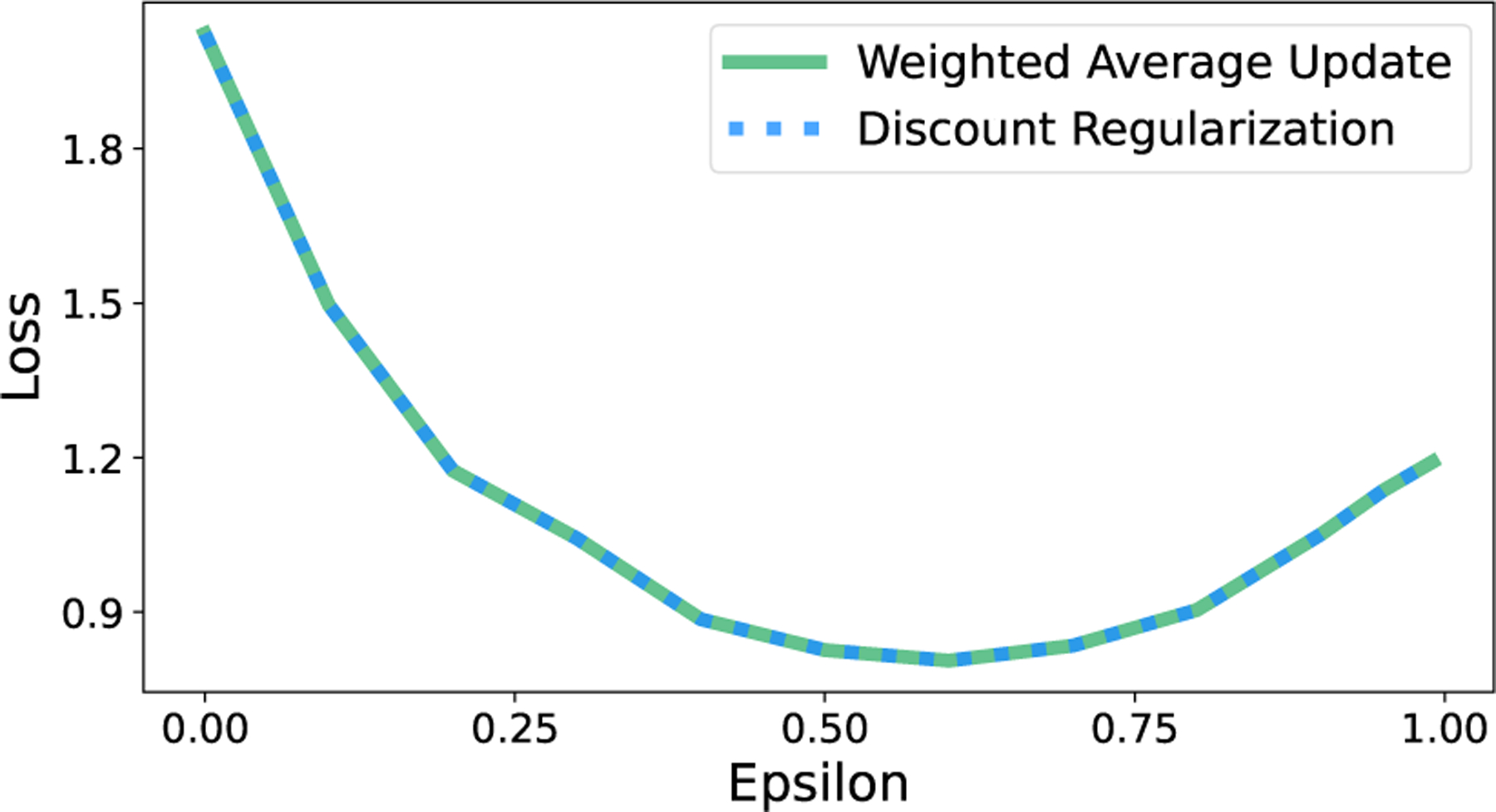
Discount regularization and a weighted average Q-function produce identical policies in model-free RL, resulting in equal loss for both methods.

As we did in the model-based case, we empirically confirm the model-free equivalence result from Thm. 2. When regularization parameter $\epsilon_{i,j}$ is the same for all state-action pairs, discount regularization and regularization using the weighted average Q-function in Eq. 4 yield the same optimal policy. Fig. 8 demonstrates that loss is identical for both methods, as is expected for identical policies.

#### 6.4.3 Results: Model-Free State-Action-Specific Regularization Underperforms Model-Based

**Figure 9:**
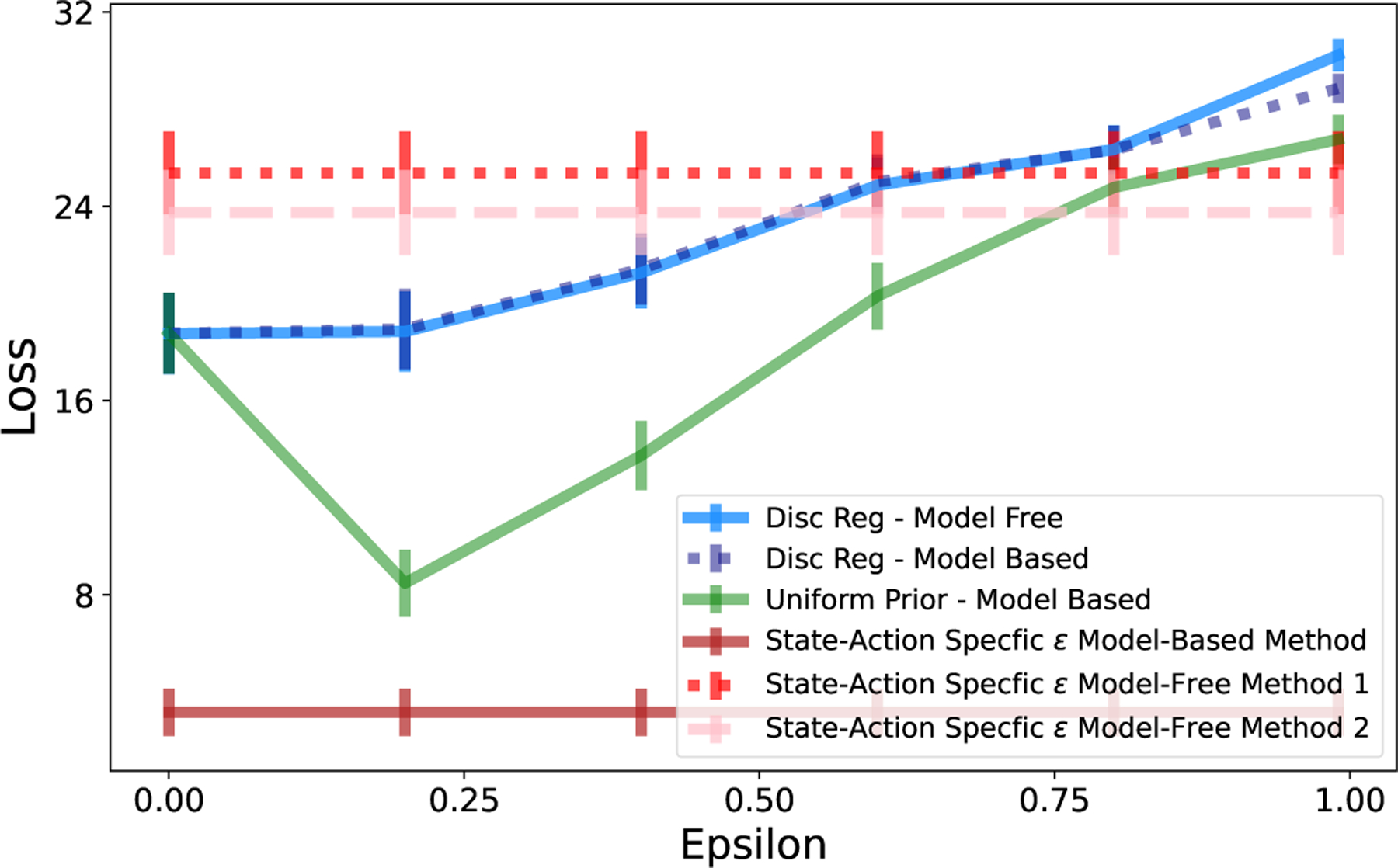
River Swim Environment. Comparison of model-free and model-based regularization methods. State-action-specific methods show loss without estimation error, using true value of T or Q.

We evaluate the loss from policies using discount regularization and using our state-action-specific regularization parameter $\epsilon_{i,j}^{\text{*}}$ for a range of environments and data set sizes. We found that our model-free method did not perform well, particularly in comparison to modelbased methods. One source of loss in the model-free method is estimation error from using the current estimate of Q in each step of FQI. To assess the best possible performance of the method, without this estimation error, we computed $\epsilon_{i,j}^{\text{*}}$ using the true values of $Q^{\pi^{\text{*}}}$. The loss for each of our methods without estimation error is illustrated in Figure 9, with results for additional data set sizes and environments are in Appendix E. Although, after removing the estimation error, our method resulted in lower loss compared to not regularizing in the 10-State Random Chain and GridWorld environments, loss was still higher than than of model-based methods.

Another cause of underperformance stems from the choice to regularize Q instead of T. The model-based method chooses the value of $\epsilon_{i,j}$ that minimizes the error between the regularized estimate of $T\left(s_{i},a_{j},\cdot \right)$ and its true value. The values for both are on the same scale: ranging from 0 to 1 and summing to 1. On the other hand, the model-free method chooses the value of $\epsilon_{i,j}$ that minimizes the error between the regularized estimate of $Q\left(s_{i},a_{j}\right)$ and its true value, which can differ significantly. These differences push the values of $\epsilon_{i,j}^{\text{*}}$ towards 0 or 1. For example, the estimated Q -values could all be higher than the true Q-values. In this case, the values of $\epsilon_{i,j}$ that minimizes the MSE in $\hat{Q}\left(s_{i},a_{j}\right)$ are all either 1 (for estimated $Q\left(s_{i},a_{j}\right)$ above than average estimate) or 0 (for estimated $Q\left(s_{i},a_{j}\right)$ below the average). Our goal in regularization is to obtain a policy that performs well in the true environment, for which we must set epsilon for all $\left(s_{i},a_{j}\right)$ together in a way that minimizes the error in *relative* values. Minimizing the absolute error for each $Q\left(s_{i},a_{j}\right)$ does not achieve this goal.

## 7 Extension to Continuous States

Theorems 1 and 2 and our resulting regularization methods apply to any number of increasingly smaller discrete states. It stands to reason, then, that we can extend our methodology to a continuous state space. We demonstrate how our state-action-specific regularization methodology can be successfully applied to a continuous state space below.

### 7.1 Methodology

We first describe how our continuous-state approach extends the ideas from the discrete-state setting, and then apply these ideas to a continuous setting where the transition function is modeled using kernel regression.

#### 7.1.1 Connection to Discrete-State Methodology

In discrete-state environments, discount regularization is commonly used with certainty-equivalence RL. Certainty-equivalence RL uses a single estimate of the transition distribution, such as the MLE, rather than modeling the uncertainty in the transition distribution. This estimate may be regularized to avoid overfitting. We showed that setting the regularization amount separately for each state-action pair can result in better-performing policies (lower loss). We extend this concept to a one-dimensional continuous state space by modeling T with a single estimate of next state given state and action, rather than using a more complex transition model that reflects the uncertainty over next state, and then regularizing this estimate before computing the optimal policy.

As discussed above, Theorems 1 and 2 imply that discount regularization pushes the values of all state-action pairs closer together. In our state-action-specific regularization method, we control the amount that the value of each state-action pair moves towards the mean separately. The same concept applies in a continuous state space. We demonstrate one way of applying these concepts to regularization with a continuous state space below.

#### 7.1.2 Kernel Regression Transition Model Method

To apply our insights from a tabular state space to a continuous state space, we take the example of modeling the transition dynamics using kernel regression and then computing the optimal policy by fitted value iteration (FVI, Szepesvári and Munos 2005). We first model the expected next state (given state and action) $s^{\prime }=E\left[T\left(s,a\right)\right]$ using a kernel regression separately for each action (specifically the Nadaraya-Watson Kernel estimator; Nadaraya 1964, Watson 1964). As in the tabular case, we regularize by planning as if the agent transitions according to any chosen distribution that is the same for all state-action pairs with probability ϵ. In that case, our regularized estimator of the expected next state given state and action is

(10) $${\hat{T}}_{\epsilon }\left(s,a_{j}\right)=\left(1-\epsilon \right)\underset{\text{kernel regression estimate}}{\underset{︸}{\frac{\sum_{d=1}^{D}K_{h}\left(s-s_{d}\right){s^{\prime }}_{d}}{\sum_{d=1}^{D}K_{h}\left(s-s_{d}\right)}}}+\underset{\begin{array}{l} \text{mean of regularization}\\ \text{transition distribution} \end{array}}{\underset{︸}{\epsilon T_{reg}}},$$

where ${\left\{s_{d},{s^{\prime }}_{d}\right\}}_{d=1}^{D}$ are the state and next state observations in the data for action $a_{j}$, $K_{h}$ is a kernel function with bandwidth h and $T_{reg}$ is the mean of the chosen regularization transition distribution.

We select $\epsilon^{\text{*}}\left(s,a_{j}\right)$, the value of ϵ that minimizes the MSE of ${\hat{T}}_{\epsilon }\left(s,a_{j}\right)$ by using the approximations of bias and variance of the Nadaraya-Watson Kernel estimator implied by the kernel regression confidence bounds presented in Wasserman (2004, p. 323). This yields the expression for $\epsilon^{\text{*}}\left(s,a_{j}\right)$ below, derived in Appendix F,

(11) $$\epsilon^{\text{*}}\left(s,a_{k}\right)=\frac{{\hat{se}}^{2}\left(s,a_{k}\right)}{{\left(T_{reg}-{\hat{T}}_{NW}\left(s,a_{k}\right)\right)}^{2}+{\hat{se}}^{2}\left(s,a_{k}\right)},$$

where:

-
$\hat{se}\left(s,a_{k}\right)=\hat{\sigma }\left(s,a_{k}\right)\sqrt{\sum_{i=1}^{D}{\left[\frac{K_{h}\left(s-s_{i}\right)}{\sum_{j=1}^{D}K_{h}\left(s-s_{j}\right)}\right]}^{2}}$

- ${\hat{\sigma }}^{2}=\frac{1}{2\left(n-1\right)}\sum_{d=1}^{D-1}{\left(s_{d+1}^{'}-s_{d}^{'}\right)}^{2}$ where the data is ordered by the values of ${\left\{s\right\}}_{d}$,
- ${\hat{T}}_{NW}$ is the Nadaraya-Watson kernel estimator for the expected next state given current state and action, ${\hat{T}}_{NW}=\frac{\sum_{d=1}^{D}K_{h}\left(s-s_{d}\right)s_{d}^{'}}{\sum_{d=1}^{D}K_{h}\left(s-s_{d}\right)}$,
- and values above are computed over the D data tuples ${\left\{s_{d},a_{k},s_{d}^{'}\right\}}_{d=1}^{D}$ separately for each given discrete action $a_{k}$.

We use this state-action-specific regularization parameter $\epsilon^{\text{*}}\left(s,a_{k}\right)$ in FVI. The procedure is detailed in Algorithm 2. We calculate $\epsilon^{\text{*}}\left(s,a_{k}\right)$ before all iterations of FVI. Then we use a weighted average Bellman update like in the tabular model-free case: using $\epsilon^{\text{*}}\left(s,a_{k}\right)$ to average the value of the next state as predicted by kernel regression and the value of the next state predicted by our fixed regularization transition distribution.

By modeling only the expected next state given state and action rather than the full distribution over next state, Algorithm 2 is more efficient than sampling-based FVI where the next state is repeatedly sampled from the model for T and $Q\left(s_{n},a_{m}\right)$ is estimated by averaging over samples. Note that line 9 of the algorithm assumes the uniform distribution over states as the regularization distribution, but this can be modified to reflect any transition distribution as appropriate for the environment.

### 7.2 Continuous-State Simulation

We demonstrate the regularization method described above on a simple continuous-state environment. The environment, simulation procedure and results are described below.


$$\begin{array}{l} \bar{\underline{\mathrm{Algorithm}2\text{ FVI with State-Action-Specific Regularization}}}\\ \text{ 1: Sample }N\text{ states}{\left\{s_{n}\right\}}_{n-1}^{N}\text{from state space}\\ \text{ 2: Initialize value function model }V_{\theta }(s)\\ \text{ 3: Learn transition model for next state given state and action }s^{\prime }|s,a=\hat{T}(s,a)\text{ using batch}\\ \text{ data and kernel regression.}\\ \text{ 4: Predict next state }s_{n,k}^{\prime }|s_{n},a_{k}\text{ for all sampled states }s_{n}\text{ and all actions }a_{k}\in A\text{ using}\\ \text{ transition model from line 3.}\\ \text{ 5: Calculate }\epsilon^{*}\left(s_{n},a_{k}\right)\text{ for all sampled states and all actions (Eq.11).}\\ \text{ 6: }\mathrm{for}\text{ i in num\_iters }\mathrm{do}\\ \text{ 7: }\mathrm{for}\text{ each sample state }{\left\{s_{n}\right\}}_{n=1}^{N}\;\mathrm{do}\\ \text{ 8: }\mathrm{for}\text{ each action }a_{k}\in A\;\mathrm{do}\\ \text{ 9: }Q\left(s_{n},a_{k}\right)=R\left(s_{n},a_{k}\right)+\gamma \left(1-\epsilon^{*}\left(s_{n},a_{k}\right)\right)V_{\theta }\left(s_{n,k}^{\prime }\right)+\gamma \epsilon^{*}\left(s_{n},a_{k}\right)\frac{1}{N}\sum_{n^{\prime }=1}^{N}V_{\theta }\left(s_{n^{\prime }}^{\prime }\right)\\ \text{10: }\mathrm{end}\;\mathrm{for}\\ \text{11: }v_{n}=\max_{k}Q\left(s_{n},a_{k}\right)\\ \text{12: }\mathrm{end}\;\mathrm{for}\\ \text{13: Update }V_{\theta }(s)\text{ using supervised learning and }{\left\{s_{n},v_{n}\right\}}_{n=1}^{N}\\ \underline{\text{14: }\mathrm{end}\;\mathrm{for}} \end{array}$$

#### 7.2.1 Environment

##### Continuous River Swim.

We demonstrate the performance of Algorithm 2 on a continuous version of the River Swim environment. We take $s\in \left[0,1\right]$ to be the one-dimensional continuous state space. We define a continuous reward function that produces a smaller positive reward near the lower end of the state space and a larger positive reward at the high end. As in the tabular version, there are two discrete actions. Action 0 moves the agent “against the current” (i.e. with more stochasticity) towards the higher reward region and action 1 moves the agent “with the current” (with less stochasticity) towards the lower reward region. Stochasticity is introduced via random uniform noise. See Appendix G for more details.

#### 7.2.2 Procedure

Like in the tabular cases, we repeatedly sample data sets of tuples $\left\{s,a,s^{\prime }\right\}$ from the true MDP. We compute the optimal policy using Algorithm 2 with either discount regularization (equivalent to a fixed value of ϵ for all states and actions) or using the state-action specific $\epsilon^{\text{*}}\left(s,a_{k}\right)$ in Eq. 11. The loss is the difference between the value of the true optimal policy and the value of the policy found using the regularized, estimated transition function, each evaluated in the true environment and averaged across sampled states. We use randomized decision trees (scikit-learn ExtraTreesRegressor; Pedregosa et al., 2011) to model value function $V\left(s\right)$.

#### 7.2.3 Results

**Figure 10:**
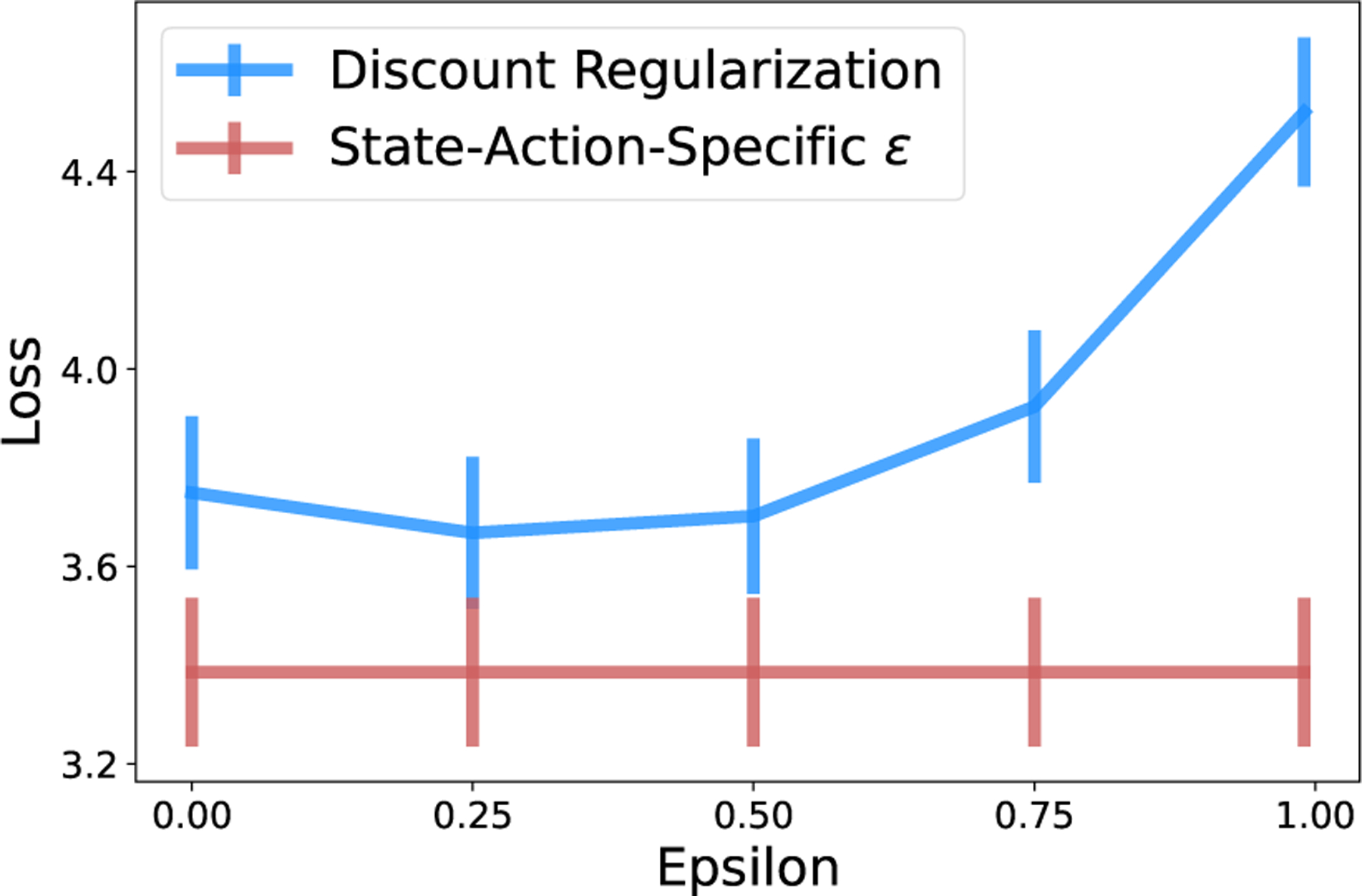
State-action specific regularization results in lower loss than discount regularization in the continuous-state River Swim environment.

Figure 10 displays the average loss across data sets for a range of values of ϵ for discount regularization, as well as loss for the state-action specific $\epsilon^{\text{*}}\left(s,a_{k}\right)$ (single loss value plotted horizontally). For this environment, the state-action specific regularization method results in lower loss than any fixed global value of ϵ. This includes outperforming the unregularized version of FVI $\left(\epsilon =0\right)$. Our state-action-specific method achieves these results with no parameter tuning or cross-validation, only a single computation for any $\left(s,a\right)$.

This method allows us to use a simple transition model with lower loss, however we note that, as discussed in the tabular case, the performance depends on the underlying transition distribution matching the chosen regularization distribution. This choice must be made carefully based on knowledge of the environment. Also, note that because our algorithm regularizes the Bellman equation directly (not the transition matrix), discount regularization and fixed ϵ weighted average generate identical policies and hence produce the same loss.

## 8 Discussion

### Comparison of Model-Based and Model-Free Methods.

The regularization methods that we introduce in this paper improve upon the performance of discount regularization, allowing us to use simple RL methods with limited data. Simulations revealed that our model-based methods were more successful than the model-free methods in low-data settings where regularization is needed. This is in agreement with the idea that model-based methods perform better in low-data settings due to their sample efficiency (Yu, 2018).

### Extension to Online Methods.

The connection between discount regularization and a weighted average transition matrix arises most obviously in the batch setting, however our methods to mitigate the pitfalls of discount regularization can be applied to online methods as well. Because the expression for $\epsilon_{i,j}^{\text{*}}$ decreases towards 0 with increasing number of transitions observed, it provides a way to decrease regularization as data is collected online. For example, we can modify the Q-learning algorithm to incorporate a weighted average update rule at each step. Please see Appendix G.1 for algorithm details and proof of concept.

### Limitations.

Our model-based method is based on learning a transition model with low MSE, which does not guarantee a good policy. In other words, it is possible to learn a good policy from a “bad” transition model and vice versa, in particular because certain errors in the model may affect the policy more than others. However, this method works well in comparison to our model-free method which directly minimizes error in Q demonstrating that minimizing the error in T rather than Q is a reasonable approach.

## 9 Conclusion

Discount regularization is a commonly used technique for dealing with noisy and sparse data. Although practitioners believe that they are simply ignoring delayed effects, we revealed deeper connections to other methods of regularization. In the model-based context, through a simple reframing of discount regularization as a weighted average transition matrix, we showed that it acts like a prior on the transition matrix that has the same distribution for all states and actions. Applying the same reframing in a model-free context revealed a connection between discount regularization and regularization methods that penalize that value function.

We showed that, problematically, discount regularization results in stronger regularization for state-action pairs with more data. To remedy the issue, we used the weighted average form to derive an explicit formula for the regularization parameter that is calculated locally for each state-action pair rather than globally. We demonstrated that this approach results in lower loss without parameter tuning in model-based RL, both with discrete or continuous states. In model-free RL, this approach was not effective, consistent with the understanding that model-based methods perform better in low-data settings.

## Figures and Tables

**Figure 11:**
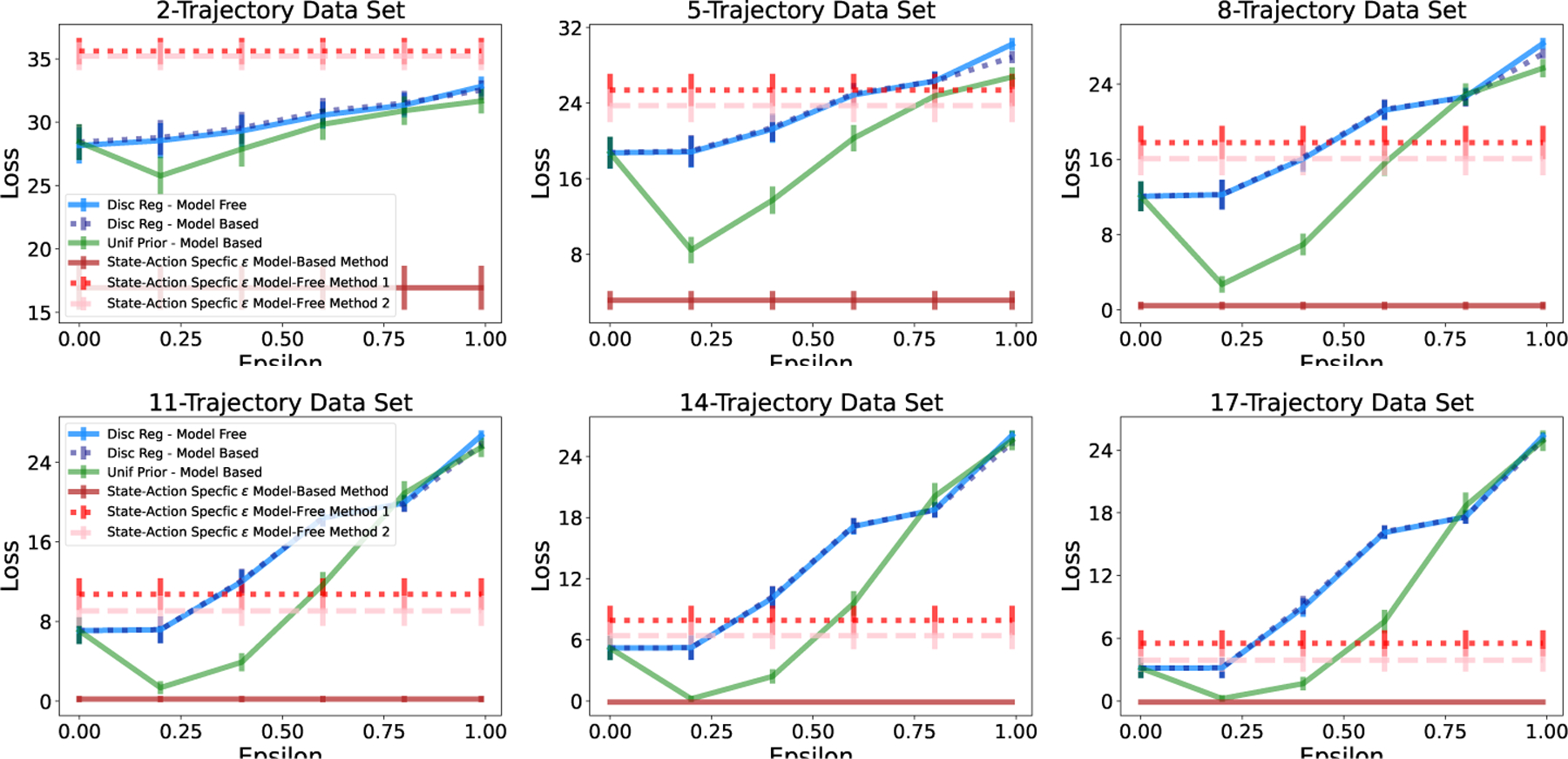
River Swim Environment. Comparison of model-free and model-based regularization methods for data sets of varying size, each with trajectories of length 20. State-action-specific methods show loss without estimation error, using true value of T or Q.

**Figure 12:**
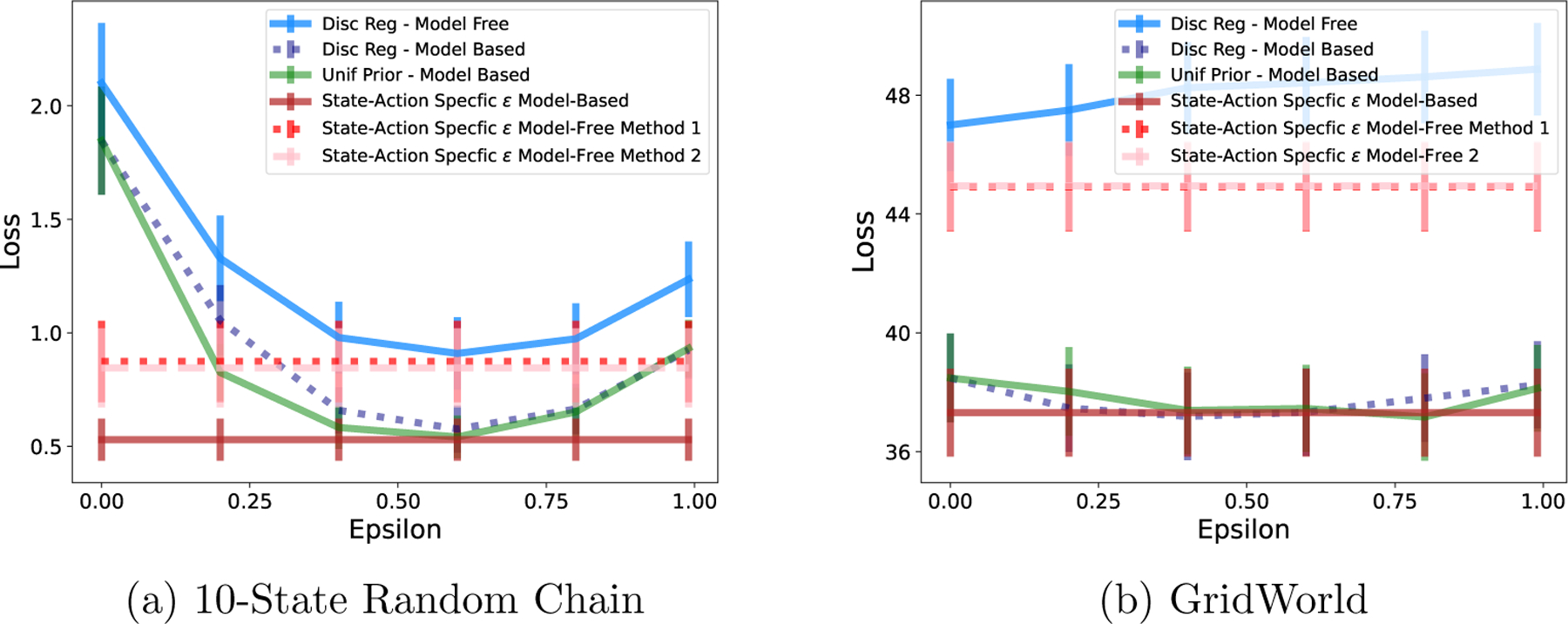
Comparison of model-free and model-based regularization methods. State-action-specific methods show loss without estimation error, using true value of T or Q

**Figure 13:**
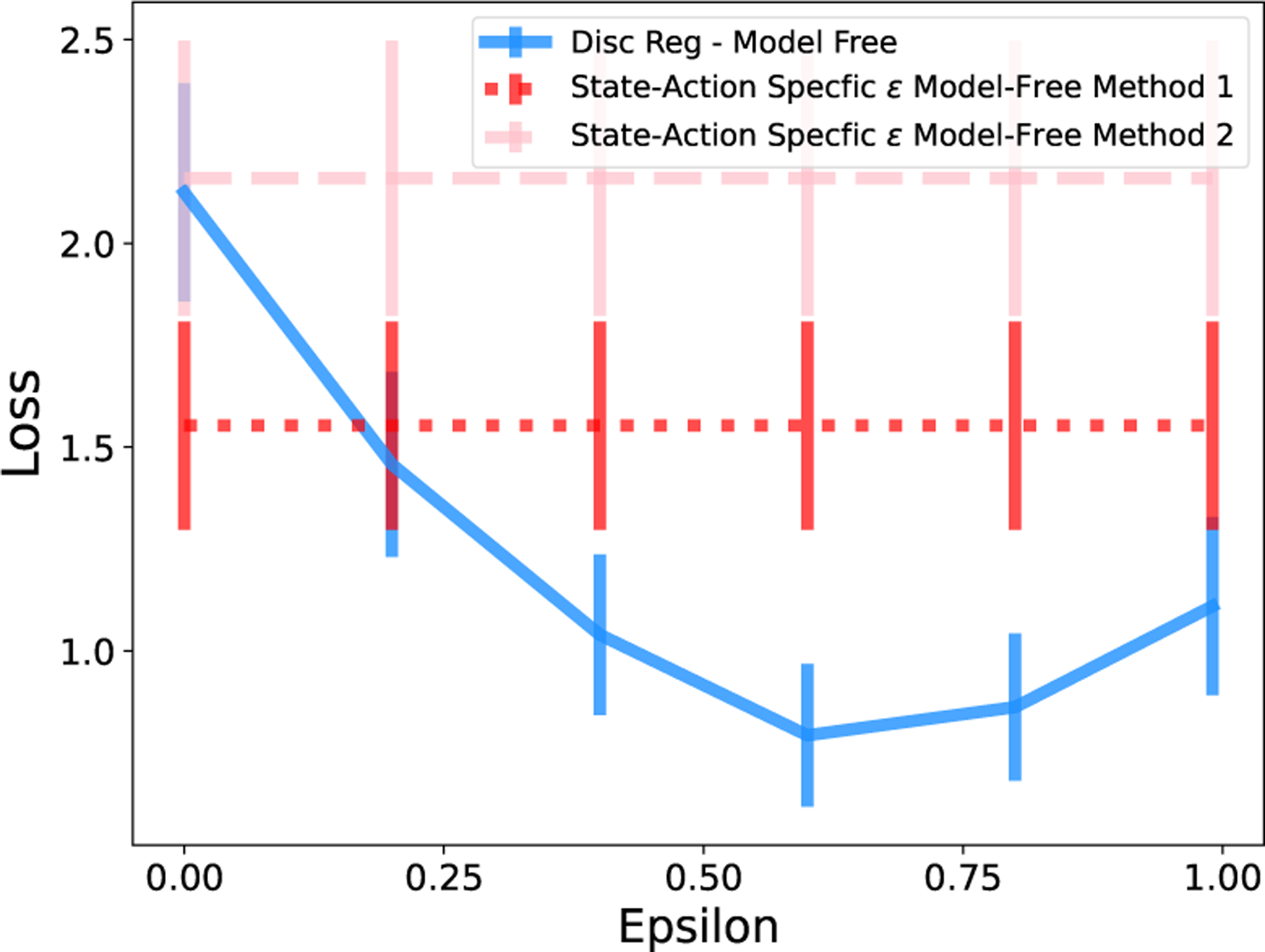
Random Chain Environment. Comparison of discount regularization and our state-action specific regularization in model-free RL.

**Figure 14:**
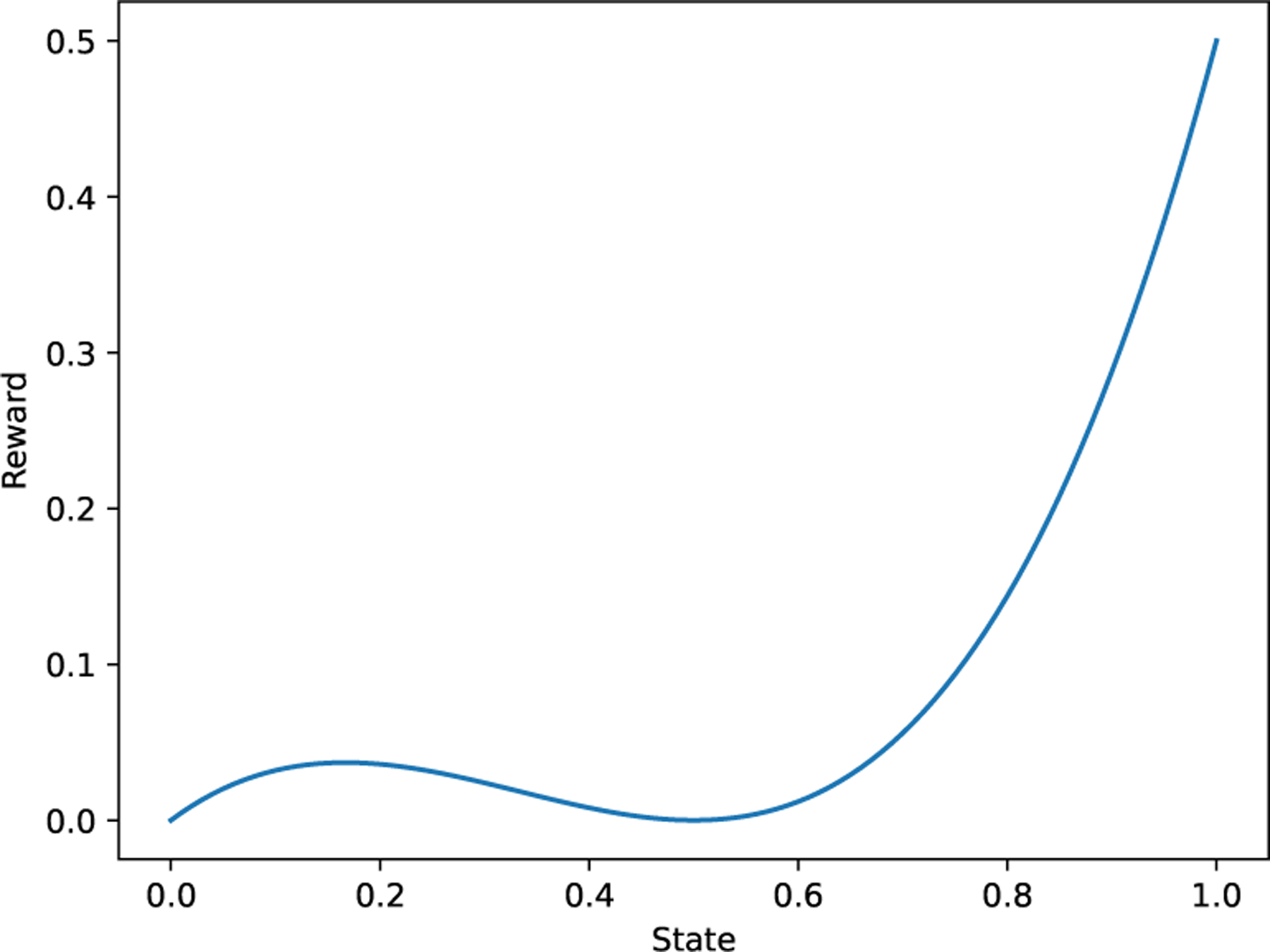
Continuous river swim environment reward function.

**Figure 15:**
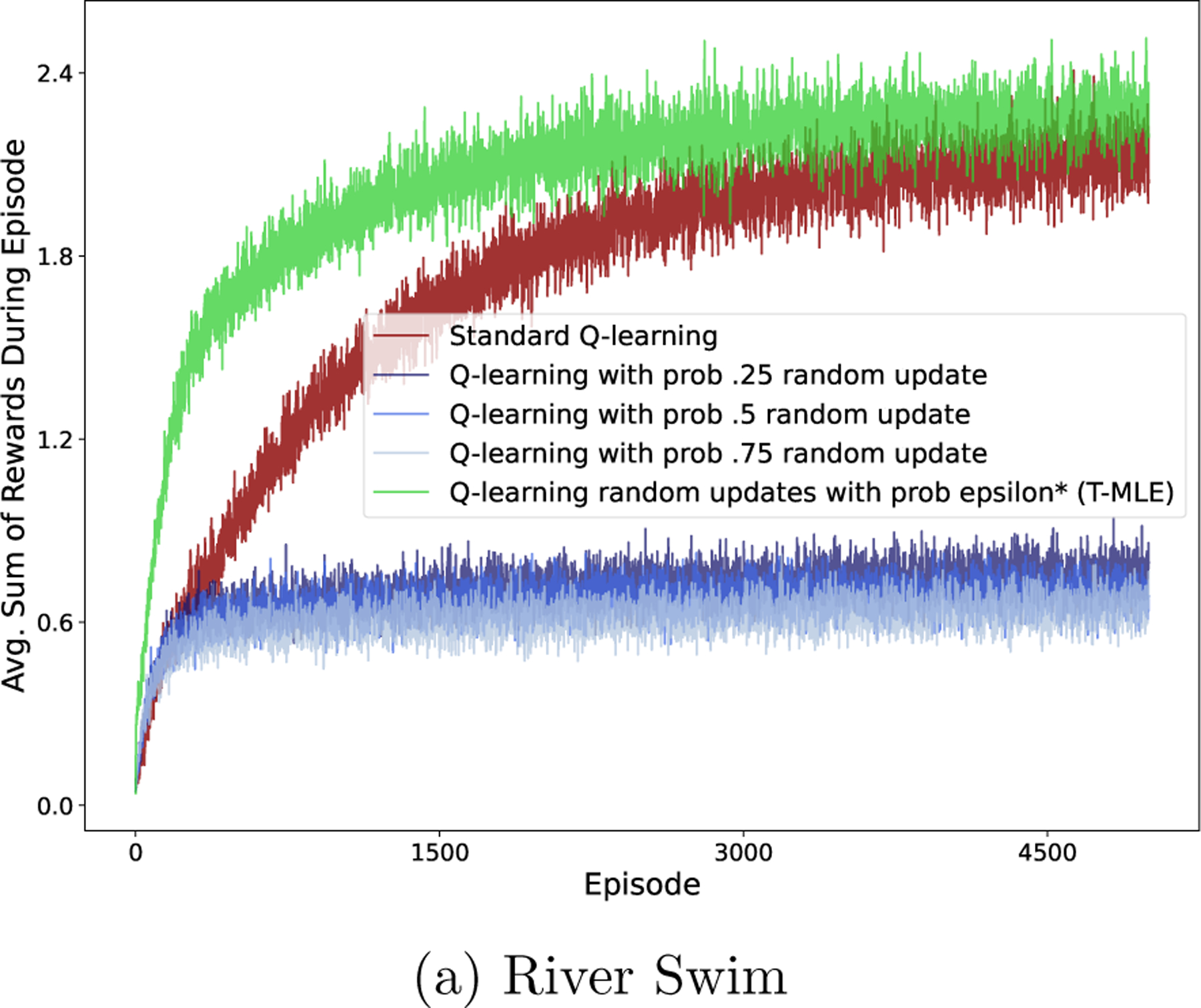
River Swim Environment. Comparison of state-action-specific regularization to standard Q-learning in our three tabular environments. We also include as a baseline our algorithm with $\epsilon^{\text{*}}$ replaced by a constant probability.

## Footnotes

1. See Appendix A.2 for details.

2. See Appendix A.1 for derivation.

3. See Appendix A.3 for details.

4. Consider, for example, the case of the River Swim Environment described in Sec. 6.1. Under the optimal policy, the agent goes right towards the large reward. Overestimating the probability of going right under the solid line action results in the agent preferring the correct action *more* and hence results in learning the correct optimal policy. Conversely, underestimating the probability of going right by a smaller amount could result in learning the wrong optimal policy despite lower MSE.

5. For simplicity, assume rewards are known.

6. Please see Def. 4.1 of Jin et al. (2021) for formal definition.

## Appendix A.

### Unified Form Derivations

#### A. 1 Dirichlet Prior Derivation

Assume prior $T_{prior}\left(s_{i},a_{j},\cdot \right)=\text{Dirichlet}\left(\langle \alpha_{i,j,1},\ldots ,\alpha_{i,j,N_{s}}\rangle \right)$ on transition matrix $T\left(s_{i},a_{j}\right)$ and let $\langle c_{i,j,1},\ldots ,c_{i,j,N_{s}}\rangle$ be the transition count data observed from state $s_{i}$ to states 1 through $N_{s}$ under action $a_{j}$. The posterior of $T\left(s_{i},a_{k},\cdot \right)$ follows a Dirichlet distribution with parameter $\langle c_{i,j,1}+\alpha_{i,j,1},\ldots ,c_{i,j,N_{s}}+\alpha_{i,j,N_{s}}\rangle$ and the posterior mean is:

$$T_{\text{post mean }}\left(s_{i},a_{j},\cdot \right)=\langle \frac{c_{i,j,1}+\alpha_{i,j,1}}{\sum_{k=1}^{N_{s}}c_{i,j,k}+\sum_{k=1}^{N_{s}}\alpha_{i,j,k}},\ldots ,\frac{c_{i,j,N_{s}}+\alpha_{i,j,N_{s}}}{\sum_{k=1}^{N_{s}}c_{i,j,k}+\sum_{k=1}^{N_{s}}\alpha_{i,j,k}}\rangle \;=\langle \frac{c_{i,j,1}}{\sum_{k=1}^{N_{s}}c_{i,j,k}+\sum_{k=1}^{N_{s}}\alpha_{i,j,k}},\ldots ,\frac{c_{i,j,N_{s}}}{\sum_{k=1}^{N_{s}}c_{i,j,k}+\sum_{k=1}^{N_{s}}\alpha_{i,j,k}}\rangle \;+\langle \frac{\alpha_{i,j,1}}{\sum_{k=1}^{N_{s}}c_{i,j,k}+\sum_{k=1}^{N_{s}}\alpha_{i,j,k}},\ldots ,\frac{\alpha_{i,j,N_{s}}}{\sum_{k=1}^{N_{s}}c_{i,j,k}+\sum_{k=1}^{N_{s}}\alpha_{i,j,k}}\rangle \;=\frac{\sum_{k=1}^{N_{s}}c_{i,j,k}}{\sum_{k=1}^{N_{s}}c_{i,j,k}}\langle \frac{c_{i,j,1}}{\sum_{k=1}^{N_{s}}c_{i,j,k}+\sum_{k=1}^{N_{s}}\alpha_{i,j,k}},\ldots ,\frac{c_{i,j,N_{s}}}{\sum_{k=1}^{N_{s}}c_{i,j,k}+\sum_{k=1}^{N_{s}}\alpha_{i,j,k}}\rangle \;+\frac{\sum_{k=1}^{N_{s}}\alpha_{i,j,k}}{\sum_{k=1}^{N_{s}}\alpha_{i,j,k}}\langle \frac{\alpha_{i,j,1}}{\sum_{k=1}^{N_{s}}c_{i,j,k}+\sum_{k=1}^{N_{s}}\alpha_{i,j,k}},\ldots ,\frac{\alpha_{i,j,N_{s}}}{\sum_{k=1}^{N_{s}}c_{i,j,k}+\sum_{k=1}^{N_{s}}\alpha_{i,j,k}}\rangle \;=\frac{\sum_{k=1}^{N_{s}}c_{i,j,k}}{\sum_{k=1}^{N_{s}}c_{i,j,k}+\sum_{k=1}^{N_{s}}\alpha_{i,j,k}}\langle \frac{c_{i,j,1}}{\sum_{k=1}^{N_{s}}c_{i,j,k}},\ldots ,\frac{c_{i,j,N_{s}}}{\sum_{k=1}^{N_{s}}c_{i,j,k}}\rangle \;+\frac{\sum_{k=1}^{N_{s}}\alpha_{i,j,k}}{\sum_{k=1}^{N_{s}}c_{i,j,k}+\sum_{k=1}^{N_{s}}\alpha_{i,j,k}}\langle \frac{\alpha_{i,j,1}}{\sum_{k=1}^{N_{s}}\alpha_{i,j,k}},\ldots ,\frac{\alpha_{i,j,N_{s}}}{\sum_{k=1}^{N_{s}}\alpha_{i,j,k}}\rangle$$

Let ${\hat{T}}_{MLE}\left(s_{i},a_{j},\cdot \right)$ be the MLE of $T\left(s_{i},a_{j},\cdot \right)$. Then,

$${\hat{T}}_{MLE}\left(s_{i},a_{j},\cdot \right)=\langle \frac{c_{i,j,1}}{\sum_{k=1}^{N_{s}}c_{i,j,k}},\ldots ,\frac{c_{i,j,N_{s}}}{\sum_{k=1}^{N_{s}}c_{i,j,k}}\rangle .$$

Let $T_{\text{prior mean}}\left(s_{i},a_{j},\cdot \right)$ be the transition matrix implied by the prior for state $s_{i}$ and action $a_{j}$, i.e. $T_{\text{prior mean }}\left(s_{i},a_{j},\cdot \right)=\langle \frac{\alpha_{i,j,1}}{\sum_{k=1}^{N_{s}}\alpha_{i,j,k}},\ldots ,\frac{\alpha_{i,j,N_{s}}}{\sum_{k=1}^{N_{s}}\alpha_{i,j,k}}\rangle$. Then we can write $T_{\text{post mean}}\left(s_{i},a_{j},\cdot \right)$ as follows.

$$T_{\text{podt mean }}\left(s_{i},a_{j},\cdot \right)=\frac{\sum_{k=1}^{N_{s}}c_{i,j,k}}{\sum_{k=1}^{N_{s}}c_{i,j,k}+\sum_{k=1}^{N_{s}}\alpha_{i,j,k}}{\hat{T}}_{MLE}\left(s_{i},a_{j},\cdot \right)\;\;+\frac{\sum_{k=1}^{N_{s}}\alpha_{i,j,k}}{\sum_{k=1}^{N_{s}}c_{i,j,k}+\sum_{k=1}^{N_{s}}\alpha_{i,j,k}}T_{\text{prior mean }}\left(s_{i},a_{j},\cdot \right)$$

Let $\epsilon =\frac{\sum_{k=1}^{N_{s}}\alpha_{i,j,k}}{\sum_{k=1}^{N_{s}}c_{i,j,k}+\sum_{k=1}^{N_{s}}\alpha_{i,j,k}}$. Then we have:

$$T_{\text{post mean }}\left(s_{i},a_{j},\cdot \right)=\left(1-\epsilon \right){\hat{T}}_{MLE}\left(s_{i},a_{j},\cdot \right)+\epsilon T_{\text{prior mean }}\left(s_{i},a_{j},\cdot \right)$$

#### A. 2 Discount Regularization

Consider the matrix form of the Bellman equation, using $\gamma_{p}$, the lower value of the discount factor used during planning for regularization: $V=R+\gamma_{p}TV$. By the steps below, we write the product $\gamma_{p}T$ from the Bellman equation as the product of true discount factor γ and a weighted average matrix.

$$\gamma_{p}T=\left[\gamma -\left(\gamma -\gamma_{p}\right)\right]T\;\left(\text{Add and subtract }\gamma \right)$$

$$\gamma_{p}T=\gamma \left(1-\frac{\left(\gamma -\gamma_{p}\right)}{\gamma }\right)T\;\left(\text{Pull out a factor of }\gamma \text{.}\right)$$

Let $T_{zeros}$ be an appropriately sized matrix of zeros. Adding $\gamma T_{zeros}$ to the right hand side does not change the equality.

$$\gamma_{p}T=\gamma \left[\left(1-\frac{\gamma -\gamma_{p}}{\gamma }\right)T+T_{zeros}\right]$$

Multiply the $T_{zeros}$ term inside the parentheses by $\frac{\gamma -\gamma_{p}}{\gamma }$. $T_{zeros}$ is all zeros so a multiplier does not affect the equality.

$$\gamma_{p}T=\gamma \left[\left(1-\frac{\gamma -\gamma_{p}}{\gamma }\right)T+\left(\frac{\gamma -\gamma_{p}}{\gamma }\right)T_{zeros}\right]$$

Let $\epsilon =\frac{\gamma -\gamma_{p}}{\gamma }$.

$$\gamma_{p}T=\gamma \left[\left(1-\epsilon \right)T_{true}+\epsilon T_{zeros}\right]$$

We have replaced the product of the planning discount factor and the true transition matrix with the product of the true discount factor and a weighted average of the transition matrix and a matrix of zeros. To put this in our framework, consider regularizing the MLE transition matrix for state-action pair $\left(s,a\right)$ via discount regularization, using planning discount factor $\gamma_{p}$. Using the proof in this section, our regularized estimated transition matrix $\hat{T}\left(s,a\right)$, is:

$$\hat{T}\left(s,a,\cdot \right)=\left(1-\epsilon \right){\hat{T}}_{MLE}\left(s,a,\cdot \right)+\epsilon T_{zeros}$$

where $\epsilon =\frac{\gamma -\gamma_{p}}{\gamma }$.

#### A. 3 Dirichlet Prior Implied by Discount Regularization

The proof of equivalence in Thm. 1 demonstrates that, for a given value of ϵ, averaging the transition matrix with either a matrix of the discrete uniform distribution or with the matrix of zeros yields the same policy. Since discount regularization applies the same ϵ to every state-action pair, the two methods are only exactly equivalent when the posterior transition matrix under a uniform prior has the same implied value of ϵ for all state-action pairs, i.e. $\epsilon =\frac{\sum_{k=1}^{N_{s}}\alpha_{i,j,k}}{\sum_{k=1}^{N_{s}}c_{i,j,k}+\sum_{k=1}^{N_{s}}\alpha_{i,j,k}}$ is the same for all state-action pairs, where $c_{i,j,k}$ and $\alpha_{i,j,k}$ are the transition count and prior from state i to state k under action j.

We can use the equivalence of the two methods for the same value of ϵ to solve for the magnitude of prior that is implied by a given discount factor in discount regularization. This is particularly interesting when $\sum_{k=1}^{N_{s}}c_{i,j,k}$ is unequal across state-action pairs $\left(s_{i},a_{j}\right)$. In this case, we can use the equivalence of discount regularization and the uniform prior to compute the different priors implied by discount regularization across state-action pairs.

Below we refer to $\sum_{k=1}^{N_{s}}c_{i,j,k}$ as $\sum c_{k}$ and $\sum_{k=1}^{N_{s}}\alpha_{i,j,k}$ as $\sum \alpha_{k}$ for brevity. Since we know both methods produce the same optimal policy for equal values of ϵ, we set the formulas for ϵ under each method equal to one another. We then solve for $\sum \alpha_{k}$ to get the magnitude of prior that is implied by a given planning discount factor $\gamma_{p}$.

$$\frac{\sum \alpha_{k}}{\sum \alpha_{k}+\sum c_{k}}=\frac{\gamma -\gamma_{p}}{\gamma }\;\;\gamma \sum \alpha_{k}=\left(\sum \alpha_{k}+\sum c_{k}\right)\left(\gamma -\gamma_{p}\right)\;\;\gamma \sum \alpha_{k}=\gamma \sum \alpha_{k}-\gamma_{p}\sum \alpha_{k}+\gamma \sum c_{k}-\gamma_{p}\sum c_{k}\;\;\gamma_{p}\sum \alpha_{k}=\sum c_{k}\left(\gamma -\gamma_{p}\right)\;\;\sum \alpha_{k}=\sum c_{k}\frac{\gamma -\gamma_{p}}{\gamma_{p}}$$

For the uniform distribution, all $\alpha_{k}$ for a given state are the same, so substitute $\sum \alpha_{k}=N\alpha_{k}$.

$$N\alpha_{k}=\sum c_{k}\frac{\gamma -\gamma_{p}}{\gamma_{p}}$$

$$\alpha_{k}=\frac{\sum c_{k}}{N_{s}}\frac{\gamma -\gamma_{p}}{\gamma_{p}}$$

So discount regularization functions like the Dirichlet prior:

(12)
$$T_{\text{prior}}\left(s_{i},a_{j},\cdot \right)∼\text{Dirichlet}\left(\frac{\sum c_{k}}{N_{s}}\frac{\gamma -\gamma_{p}}{\gamma_{p}},\ldots ,\frac{\sum c_{k}}{N_{s}}\frac{\gamma -\gamma_{p}}{\gamma_{p}}\right)$$

where again $\sum c_{k}$ is the total number of transitions observed in the data starting at state $s_{i}$ under action $a_{j}$.

## Appendix B.

### Proof of Thm. 1 Step 1

Suppose $Q_{1}^{\text{*}}\left(s,a\right)$ is the unique solution to:

(a)
$$Q_{1}^{\text{*}}\left(s,a\right)=R\left(s,a\right)+c+\gamma \sum_{s^{\prime }}T\left(s,a,s^{\prime }\right)\underset{a^{\prime }}{\text{max}}Q_{1}^{\text{*}}\left(s^{\prime },a^{\prime }\right)$$

and $Q_{2}^{\text{*}}\left(s,a\right)$ is the unique solution to:

(b)
$$Q_{2}^{\text{*}}\left(s,a\right)=R\left(s,a\right)+\gamma \sum_{s^{\prime }}T\left(s,a,s^{\prime }\right)\underset{a^{\prime }}{\text{max}}Q_{s}^{\text{*}}\left(s^{\prime },a^{\prime }\right)$$

First note that $Q_{1}^{\text{*}}\left(s,a\right)={\text{max}}_{\pi }E_{T,\pi }\left[\sum_{t=0}^{\infty }\gamma^{t}\left(R\left(S_{t},A_{t}\right)+c\right)|S_{0}=s,A_{0}=a\right]$ is the unique solution to Eq. (a) and $Q_{2}^{\text{*}}\left(s,a\right)={\text{max}}_{\pi }E_{T,\pi }\left[\sum_{t=0}^{\infty }\gamma^{t}R\left(S_{t},A_{t}\right)|S_{0}=s,A_{0}=a\right]$ is the unique solution to Eq. (b).

But $Q_{1}^{\text{*}}\left(s,a\right)={\text{max}}_{\pi }E_{T,\pi }\left[\sum_{t=0}^{\infty }\gamma^{t}R\left(S_{t},A_{t}\right)|S_{0}=s,A_{0}=a\right]+\sum_{t=0}^{\infty }\gamma^{t}c=Q_{2}^{\text{*}}\left(s,a\right)+\frac{c}{1-\gamma }$. Thus $Q_{1}^{\text{*}}$ and $Q_{2}^{\text{*}}$ have the same argmax and correspond to the same optimal policy, $\pi_{1}^{\text{*}}=\pi_{2}^{\text{*}}$.

## Appendix C.

### Uniform Prior MSE Calcuation

Let $c_{i,j}=\sum_{k=1}^{N_{s}}c_{i,j,k}$ be the transition observation count for state-action pair $\left(s_{i},a_{j}\right)$ in the data. Let $T\left(s_{i},a_{j},\cdot \right)$ be the transition probability distribution under action $a_{j}$ starting at state $s_{i}$. $N_{s}$ is the number of states in the MDP.

$$\text{MSE}\left(\hat{T}\left(s_{i},a_{j},\cdot \right)\right)=\sum_{k=1}^{N_{s}}\left(\text{Variance}\left(\hat{T}\left(s_{i},a_{j},s_{k}\right)\right)+{\text{Bias}}^{2}\left(\hat{T}\left(s_{i},a_{j},s_{k}\right)\right)\right)$$

$$\text{Variance}\left({\hat{T}}_{\text{unif}}\left(s_{i},a_{j},s_{k}\right)\right)=\text{Variance}\left(\left(1-\epsilon_{i,j}\right){\hat{T}}_{\text{MLE}}\left(s_{i},a_{j},s_{k}\right)+\epsilon_{i,j}\frac{1}{N_{s}}\right)\;\;=\text{Variance}\left(\left(1-\epsilon_{i,j}\right){\hat{T}}_{\text{MLE}}\left(s_{i},a_{j},s_{k}\right)\right)+\text{Variance}\left(\epsilon_{i,j}\frac{1}{N_{s}}\right)\;\;={\left(1-\epsilon_{i,j}\right)}^{2}\text{Variance}\left({\hat{T}}_{\text{MLE}}\left(s_{i},a_{j},s_{k}\right)\right)\;\;={\left(1-\epsilon_{i,j}\right)}^{2}\frac{1}{c_{i\cdot j}}T\left(s_{i},a_{j},s_{k}\right)\left(1-T\left(s_{i},a_{j},s_{k}\right)\right)$$

$$\text{Bias}\left({\hat{T}}_{\text{unif}}\left(s_{i},a_{j},s_{k}\right)\right)=E\left[{\hat{T}}_{\text{unif}}\left(s_{i},a_{j},s_{k}\right)-T\left(s_{i},a_{j},s_{k}\right)\right]\;\;=E\left[\left(1-\epsilon_{i,j}\right){\hat{T}}_{\text{MLE}}\left(s_{i},a_{j},s_{k}\right)+\epsilon_{i,j}\frac{1}{N_{s}}-T\left(s_{i},a_{j},s_{k}\right)\right]\;\;=\left(1-\epsilon_{i,j}\right)T\left(s_{i},a_{j},s_{k}\right)+\epsilon_{i,j}\frac{1}{N_{s}}-T\left(s_{i},a_{j},s_{k}\right)\;\;=\epsilon_{i,j}\left(\frac{1}{N_{s}}-T\left(s_{i},a_{j},s_{k}\right)\right)$$

$$\text{MSE}\left({\hat{T}}_{\text{unif}}\left(s_{i},a_{j},\cdot \right)\right)=\sum_{k=1}^{N_{s}}\left[{\left(1-\epsilon_{i,j}\right)}^{2}\frac{1}{c_{i\cdot j}}T\left(s_{i},a_{j},s_{k}\right)\left(1-T\left(s_{i},a_{j},s_{k}\right)\right)+\epsilon_{i,j}^{2}{\left(\frac{1}{N_{s}}-T\left(s_{i},a_{j},s_{k}\right)\right)}^{2}\right]$$

To solve for the value of $\epsilon_{i,j}$ that minimizes the MSE, set the first derivative equal to 0.

$$\frac{\partial \text{MSE}}{\epsilon_{i,j}}=\sum_{k=1}^{N_{s}}-2\left(1-\epsilon_{i,j}\right)\frac{1}{c_{i\cdot j}}T\left(s_{i},a_{j},s_{k}\right)\left(1-T\left(s_{i},a_{j},s_{k}\right)\right)\;\;+2\epsilon_{i,j}{\left(\frac{1}{N_{s}}-T\left(s_{i},a_{j},s_{k}\right)\right)}^{2}=0$$

$$\sum_{j=1}^{N_{s}}-2\frac{1}{c_{i.j}}T\left(s_{i},a_{j},s_{k}\right)\left(1-T\left(s_{i},a_{j},s_{k}\right)\right)+2\epsilon_{i,j}\frac{1}{c_{i.j}}T\left(s_{i},a_{j},s_{k}\right)\left(1-T\left(s_{i},a_{j},s_{k}\right)\right)\;\;+2\epsilon_{i,j}{\left(\frac{1}{N_{s}}-T\left(s_{i},a_{j},s_{k}\right)\right)}^{2}=0$$

$$\sum_{k=1}^{N_{s}}\epsilon_{i,j}\left[\frac{1}{c_{i\cdot j}}T\left(s_{i},a_{j},s_{k}\right)\left(1-T\left(s_{i},a_{j},s_{k}\right)\right)+{\left(\frac{1}{N_{s}}-T\left(s_{i},a_{j},s_{k}\right)\right)}^{2}\right]\;\;=\sum_{k=1}^{N_{s}}\frac{1}{c_{i.j}}T\left(s_{i},a_{j},s_{k}\right)\left(1-T\left(s_{i},a_{j},s_{k}\right)\right)$$

$$\epsilon_{i,j}=\frac{\sum_{k=1}^{N_{s}}\frac{1}{c_{i,j}}T\left(s_{i},a_{j},s_{k}\right)\left(1-T\left(s_{i},a_{j},s_{k}\right)\right)}{\sum_{k=1}^{N_{s}}\frac{1}{c_{i,j}}T\left(s_{i},a_{j},s_{k}\right)\left(1-T\left(s_{i},a_{j},s_{k}\right)\right)+{\left(\frac{1}{N_{s}}-T\left(s_{i},a_{j},s_{k}\right)\right)}^{2}}\;\;=\frac{\sum_{k=1}^{N_{s}}T\left(s_{i},a_{j},s_{k}\right)\left(1-T\left(s_{i},a_{j},s_{k}\right)\right)}{\sum_{k=1}^{N_{k}}T\left(s_{i},a_{j},s_{k}\right)\left(1-T\left(s_{i},a_{j},s_{k}\right)\right)+c_{i,j}{\left(\frac{1}{N_{s}}-T\left(s_{i},a_{j},s_{k}\right)\right)}^{2}}\;\;=\frac{\sum_{k=1}^{N_{s}}\left[T\left(s_{i},a_{j},s_{k}\right)\left(1-T\left(s_{i},a_{j},s_{k}\right)\right)\right]/\sum_{k=1}^{N_{s}}\left[{\left(\frac{1}{N_{s}}-T\left(s_{i},a_{j},s_{k}\right)\right)}^{2}\right]}{\sum_{k=1}^{N_{s}}\left[T\left(s_{i},a_{j},s_{k}\right)\left(1-T\left(s_{i},a_{j},s_{k}\right)\right)\right]/\sum_{k=1}^{N_{s}}\left[{\left(\frac{1}{N_{s}}-T\left(s_{i},a_{j},s_{k}\right)\right)}^{2}\right]+c_{i,j}}$$

## Appendix D.

### Model Free State-action-specific Regularization Calculations

We calculate mean squared error in value $Q\left(s,a\right)$ for for given state-action pair $\left(s,a\right)$ with fixed policy π—then solve for the value of ϵ that minimizes error.

#### D. 1 Definitions

We define the following variables:

- $N_{s}$: number of states
- $N_{a}$: number of actions
- C: Count data matrix, dimension $N_{s}N_{a}\times N_{s}$ (one row for every $\left(\text{s,a}\right)$, one column for every s’)
- $Q^{\pi }$: Matrix of state-action values, $N_{s}N_{a}\times 1$ (one row for every $\left(\text{s,a}\right)$)
- **1**: A vector of ones of appropriate size
- Π: matrix mapping $Q^{\pi }$ to $V^{\pi }$ based on policy π, dimension $N_{s}N_{a}\times N_{s}$. There is a column for each state and a row for each state-action pair. For each state column, the row corresponding to the state-action pair of the optimal action gets a 1, all others 0.
- $\text{Diag}\left(ℰ\right)$: $N_{s}N_{a}\times N_{s}N_{a}$ matrix with $\epsilon \left(s,a\right)$ across main diagonal, 0s elsewhere.
- $v^{\pi }$: $N_{s}\times 1$ vector of state values under policy π, equal to ${\text{Π}}^{T}Q^{\pi }$
- ${\left[{\text{Π}}^{T}Q^{\pi }\right]}_{sq}$: $N_{s}\times 1$ elementwise square of ${\text{Π}}^{T}Q^{\pi }$, the value under policy π

#### D. 2 State-Action-Specific Regularization Parameter Calculation Method 1

The first way we calculate the value of $\epsilon^{\text{*}}\left(s,a\right)$ to use at each iteration of our modified fitted Q-iteration (Algo 1) is to calculate the sum of squared errors across all transition data $\left(s,a,s^{\prime }\right)$ in the batch data set, then solve for the value of ϵ that minimizes the SSE.

$$SSE=\sum_{i,j}\sum_{k=1}^{N_{s}}\sum_{s^{\prime }|\left(s_{i},a_{j}\right)\text{ in data}}𝟙\left\{s_{\text{data }=s_{k}}^{'}\right\}\left(R\left(s_{i},a_{j}\right)+\gamma \left(1-\epsilon_{i,j}\right)Q^{\pi }\left(s_{k},\pi \left(s_{k}\right)\right)\right.\;\;+\gamma \epsilon_{i,j}\frac{1}{N_{s}}\sum_{k^{\prime }=1}^{N_{s}}Q^{\pi }\left(s_{k^{\prime }},\pi \left(s_{k^{\prime }}\right)\right)-Q^{\pi }\left(s_{i},a_{j}\right){)}^{2}\;\;=\sum_{i,j}\sum_{k=1}^{N_{s}}c_{i,j,k}\left(R\left(s_{i},a_{j}\right)+\gamma \left(1-\epsilon_{i,j}\right)Q^{\pi }\left(s_{k},\pi \left(s_{k}\right)\right)\right.\;\;+\gamma \epsilon_{i,j}\frac{1}{N_{s}}\sum_{k^{\prime }=1}^{N_{s}}Q^{\pi }\left(s_{k^{\prime }},\pi \left(s_{k^{\prime }}\right)\right)-Q^{\pi }\left(s_{i},a_{j}\right){)}^{2}$$

Set the derivative with respect to $\epsilon_{i,j}$ for one specific $\left(\text{i,j}\right)$ equal to 0. Call the state-action pair that the differentiation is with respect to $\left(i^{\text{*}},j^{\text{*}}\right)$.

$$\frac{\partial SSE}{\epsilon_{i^{\text{*}},j^{\text{*}}}}=2\sum_{k=1}^{N_{s}}\left[c_{i^{\text{*}},j^{\text{*}},k}\left\{R\left(s_{i^{\text{*}}},a_{j^{\text{*}}}\right)+\gamma \left(1-\epsilon_{i^{\text{*}},j^{\text{*}}}\right)Q^{\pi }\left(s_{k},\pi \left(s_{k}\right)\right)\right.\right.\;\;+\epsilon_{i^{\text{*}},j^{\text{*}}}\frac{\gamma }{N_{s}}\underset{\text{constant}\forall \left(s_{i^{\text{*}}},a_{j^{\text{*}}}\right)}{\underset{︸}{\sum_{k^{\prime }=1}^{N_{s}}Q^{\pi }\left(s_{k^{\prime }},\pi \left(s_{k^{\prime }}\right)\right)}}-Q^{\pi }\left(s_{i^{\text{*}}},a_{j^{\text{*}}}\right)\}\left\{-\gamma Q^{\pi }\left(s_{k},\pi \left(s_{k}\right)\right)\right.\;\;+\frac{\gamma }{N_{s}}\underset{\text{constant}\forall \left(s^{\text{*}},a^{\text{*}}\right)}{\underset{︸}{\sum_{k^{\prime }=1}^{N_{s}}Q^{\pi }\left(s_{k^{\prime }},\pi \left(s_{k^{\prime }}\right)\right)}}\}]=0$$

$\frac{1}{N_{s}}\sum_{k^{\prime }=1}^{N_{s}}Q^{\pi }\left(s_{k^{\prime }},\pi \left(s_{k^{\prime }}\right)\right)$, the average value across states under policy π, is constant, so replace with $v_{avg}^{\pi }$.

$$0=\sum_{k=1}^{N_{s}}\left[c_{i^{\text{*}},j^{\text{*}},k}\left(-\gamma Q^{\pi }\left(s_{k},\pi \left(s_{k}\right)\right)+\gamma v_{\text{avg }}^{\pi }\right)\right.\;\;\left.\left(R\left(s_{i^{\text{*}}},a_{j^{\text{*}}}\right)+\gamma \left(1-\epsilon_{i^{\text{*}},j^{\text{*}}}\right)Q^{\pi }\left(s_{k},\pi \left(s_{k}\right)\right)+\epsilon_{i^{\text{*}},j^{\text{*}}}\gamma v_{avg}^{\pi }-Q^{\pi }\left(s_{i^{\text{*}}},a_{j^{\text{*}}}\right)\right)\right]$$

Multiply out the terms.

$$0=-\gamma R\left(s_{i^{\text{*}}},a_{j^{\text{*}}}\right)\sum_{k=1}^{N_{s}}c_{i^{\text{*}},j^{\text{*}},k}Q^{\pi }\left(s_{k},\pi \left(s_{k}\right)\right)-\gamma^{2}\left(1-\epsilon_{i^{\text{*}},j^{\text{*}}}\right)\sum_{k=1}^{N_{s}}c_{i^{\text{*}},j^{\text{*}},k}Q^{\pi }{\left(s_{k},\pi \left(s_{k}\right)\right)}^{2}\;-\gamma^{2}v_{avg}^{\pi }\epsilon_{i^{\text{*}},j^{\text{*}}}\sum_{k=1}^{N_{s}}c_{i^{\text{*}},j^{\text{*}},k}Q^{\pi }\left(s_{k},\pi \left(s_{n}\right)\right)+\gamma Q^{\pi }\left(s_{i^{\text{*}}},a_{j^{\text{*}}}\right)\sum_{k=1}^{N_{s}}c_{i^{\text{*}},j^{\text{*}},k}Q^{\pi }\left(s_{k},\pi \left(s_{k}\right)\right)\;+\gamma v_{avg}^{\pi }R\left(s_{i^{\text{*}}},a_{j^{\text{*}}}\right)\sum_{k=1}^{N_{s}}c_{i^{\text{*}},j^{\text{*}},k}+\gamma^{2}v_{avg}^{\pi }\left(1-\epsilon_{i^{\text{*}},j^{\text{*}}}\right)\sum_{k=1}^{N_{s}}c_{i^{\text{*}},j^{\text{*}},k}Q^{\pi }\left(s_{k},\pi \left(s_{k}\right)\right)\;+\gamma^{2}{\left(v_{avg}^{\pi }\right)}^{2}\epsilon_{i^{\text{*}},j^{\text{*}}}\sum_{k=1}^{N_{s}}c_{i^{\text{*}},j^{\text{*}},k}-\gamma v_{avg}^{\pi }Q^{\pi }\left(s_{i^{\text{*}}},a_{j^{\text{*}}}\right)\sum_{k=1}^{N_{s}}c_{i^{\text{*}},j^{\text{*}},k}$$

Write for all $\left(\text{s,a}\right)$ in matrix form. We use the following notation for the matrix equation: ℰ is an $N_{s}N_{a}\times N_{s}N_{a}$ matrix with $\epsilon_{i,j}$ across the main diagonal, C is the $N_{s}N_{a}\times N_{s}$ matrix of transition counts (one row for every $\left(s,a\right)$, one column for every $s^{\prime }$), Π is a matrix mapping $Q^{\pi }$ to $V^{\pi }$ for fixed policy π.

$$\vec{0}=-\gamma Diag\left(R\right)C{\text{Π}}^{T}Q^{\pi }-\gamma^{2}Diag\left(C\underset{\text{elementwise square}}{\underset{︸}{{\left[{\text{Π}}^{T}Q^{\pi }\right]}_{sq}}}\right)\left(1_{N_{s}N_{a}\times 1}-ℰ\right)\;-\gamma^{2}v_{avg}^{\pi }Diag\left(C{\text{Π}}^{T}Q^{\pi }\right)ℰ+\gamma Diag\left(C{\text{Π}}^{T}Q^{\pi }\right)Q^{\pi }\;+\gamma v_{avg}^{\pi }Diag\left(R\right)C1_{N_{s}\times 1}+\gamma^{2}kv_{avg}^{\pi }Diag\left(C{\text{Π}}^{T}Q^{\pi }\right)\left(1_{N_{s}N_{a}\times 1}-ℰ\right)\;+\gamma^{2}{\left(v_{avg}^{\pi }\right)}^{2}Diag\left(C1_{N_{s}\times 1}\right)ℰ-\gamma v_{avg}^{\pi }Diag\left(C1_{N_{s}\times 1}\right)Q^{\pi }$$

Solve for ℰ.

$$\begin{array}{l} \gamma Diag\left(R\right)C{\text{Π}}^{T}Q^{\pi }+\gamma^{2}Diag\left(C\underset{\text{elementwise square}}{\underset{︸}{{\left[{\text{Π}}^{T}Q^{\pi }\right]}_{sq}}}\right)1_{N_{s}N_{a}\times 1}-\gamma Diag\left(C{\text{Π}}^{T}Q^{\pi }\right)Q^{\pi }\\ -\gamma v_{avg}^{\pi }Diag\left(R\right)C1_{N_{s}\times 1}-\gamma^{2}v_{avg}^{\pi }Diag\left(C{\text{Π}}^{T}Q^{\pi }\right)1_{N_{s}N_{a}\times 1}+\gamma v_{avg}^{\pi }Diag\left(C1_{N_{s}\times 1}\right)Q^{\pi }\\ =\gamma^{2}Diag\left(C\underset{\text{elementwise square}}{\underset{︸}{{\left[{\text{Π}}^{T}Q^{\pi }\right]}_{sq}}}\right)ℰ-\gamma^{2}v_{avg}^{\pi }Diag\left(C{\text{Π}}^{T}Q^{\pi }\right)ℰ-\gamma^{2}v_{avg}^{\pi }Diag\left(C{\text{Π}}^{T}Q^{\pi }\right)ℰ\\ +\gamma^{2}{\left(v_{avg}^{\pi }\right)}^{2}Diag\left(C1_{N_{s}\times 1}\right)ℰ \end{array}$$

Pull out factor of ℰ

$$\begin{array}{l} \gamma Diag\left(R\right)C{\text{Π}}^{T}Q^{\pi }+\gamma^{2}Diag\left(C\underset{\text{elementwise square}}{\underset{︸}{{\left[{\text{Π}}^{T}Q^{\pi }\right]}_{sq}}}\right)1_{N_{s}N_{a}\times 1}-\gamma Diag\left(C{\text{Π}}^{T}Q^{\pi }\right)Q^{\pi }\\ -\gamma v_{avg}^{\pi }Diag\left(R\right)C1_{N_{s}\times 1}-\gamma^{2}v_{avg}^{\pi }Diag\left(C{\text{Π}}^{T}Q^{\pi }\right)1_{N_{s}N_{a}\times 1}+\gamma v_{avg}^{\pi }Diag\left(C1_{N_{s}\times 1}\right)Q^{\pi }\\ =\left[\gamma^{2}Diag\left(C\underset{\text{elementwise square}}{\underset{︸}{{\left[{\text{Π}}^{T}Q^{\pi }\right]}_{sq}}}\right)\right.-\gamma^{2}v_{avg}^{\pi }Diag\left(C{\text{Π}}^{T}Q^{\pi }\right)\\ \left.-\gamma^{2}v_{avg}^{\pi }Diag\left(C{\text{Π}}^{T}Q^{\pi }\right)+\gamma^{2}{\left(v_{avg}^{\pi }\right)}^{2}Diag\left(C1_{N_{s}\times 1}\right)\right]ℰ \end{array}$$

$$ℰ={\left[\gamma^{2}Diag\left(C\underset{\text{elementwise square}}{\underset{︸}{{\left[{\text{Π}}^{T}Q^{\pi }\right]}_{sq}}}\right)-2\gamma^{2}v_{avg}^{\pi }Diag\left(C{\text{Π}}^{T}Q^{\pi }\right)+\gamma^{2}{\left(v_{avg}^{\pi }\right)}^{2}Diag\left(C1_{N_{s}\times 1}\right)\right]}^{-1}\;\left[\gamma Diag\left(R\right)C{\text{Π}}^{T}Q^{\pi }+\gamma^{2}Diag\left(C\underset{\text{elementwise square}}{\underset{︸}{{\left[{\text{Π}}^{T}Q^{\pi }\right]}_{sq}}}\right)1_{N_{s}N_{a}\times 1}-\gamma Diag\left(C{\text{Π}}^{T}Q^{\pi }\right)Q^{\pi }\right.\;\left.-\gamma v_{avg}^{\pi }Diag\left(R\right)C1_{N_{s}\times 1}-\gamma^{2}v_{avg}^{\pi }Diag\left(C{\text{Π}}^{T}Q^{\pi }\right)1_{N_{s}N_{a}\times 1}+\gamma v_{avg}^{\pi }Diag\left(C1_{N_{s}\times 1}\right)Q^{\pi }\right]$$

Now we have an expression for ϵ in terms of $Q^{\pi }$. Algorithm 1 alternates between updating $Q\left(s,a\right)$ by linear regression and updating $\epsilon_{i,j}^{\text{*}}$ by the equation above.

#### D. 3 State-Action-Specific Regularization Parameter Calculation Method 2

As an alternative method, we also calculated ℰ using the assumption that the count data follows a multinomial distribution, as we did in the model-based case.

$$\text{True Q}:Q^{\pi }\left(s_{i},a_{j}\right)=R\left(s_{i},a_{j}\right)+\gamma \sum_{k=1}^{N_{s}}T\left(s_{j},a_{j},s_{k}\right)v^{\pi }\left(s_{k}\right)$$

$$\text{Reqularized Q}:Q_{\epsilon }^{\pi }\left(s_{i},a_{j}\right)=R\left(s_{i},a_{j}\right)+\gamma \left(1-\epsilon_{i,j}\right)\sum_{k=1}^{N_{s}}T\left(s_{i},a_{j},s_{k}\right)V^{\pi }\left(s_{k}\right)+\gamma \epsilon_{i,j}\frac{1}{N_{s}}\sum_{k=1}^{N_{s}}V^{\pi }\left(s_{k}\right)$$

$\text{Variance}\left({\hat{Q}}_{\epsilon }^{\pi }\left(s_{i},a_{j}\right)\right)$: Consider fixed $V^{\pi }$. Then,

$$\text{Var}\left({\hat{Q}}_{\epsilon }^{\pi }\left(s_{i},a_{j}\right)\right)=\text{Var}\left(\gamma \left(1-\epsilon_{i,j}\right)\sum_{k=1}^{N_{s}}\hat{T}\left(s_{i},a_{j},s_{k}\right)V^{\pi }\left(s_{k}\right)\right)\;\;=\text{Var}\left(\gamma \left(1-\epsilon_{i,j}\right)\sum_{k=1}^{N_{s}}\frac{c_{i,j,k}}{\sum_{k^{\prime }}c_{i,j,k^{\prime }}}V^{\pi }\left(s_{k}\right)\right.\;\;=\frac{\gamma^{2}{\left(1-\epsilon_{i,j}\right)}^{2}}{{\left(\sum_{k^{\prime }=1}^{N_{s}}c_{i,j,k^{\prime }}\right)}^{2}}\text{Var}\left(\sum_{k=1}^{N_{s}}c_{i,j,k}V^{\pi }\left(s_{k}\right)\right)$$

Expand using the variance and covariance of a multinomial distribution. Let $c_{i,j}=\sum_{k=1}^{N_{s}}c_{i,j,k}$, the number of transition counts in the data starting at state $s_{i}$, taking action $a_{k}$, and transitioning to any state.

$$=\frac{\gamma^{2}{\left(1-\epsilon_{i,j}\right)}^{2}}{c_{i,j}^{2}}\left[\sum_{k=1}^{N_{s}}{\left(V^{\pi }\left(s_{k}\right)\right)}^{2}c_{i,j}T\left(s_{i},a_{j},s_{k}\right)\left(1-T\left(s_{i},a_{j},s_{k}\right)\right)\right.\;\left.-2\sum_{k=1}^{N_{s}}\sum_{k^{\prime }\neq k}c_{i,j}V^{\pi }\left(s_{k}\right)V^{\pi }\left(s_{k^{\prime }}\right)T\left(s_{i},a_{j},s_{k}\right)T\left(s_{i},a_{j},s_{k^{\prime }}\right)\right]\;=\frac{\gamma^{2}{\left(1-\epsilon_{i,j}\right)}^{2}}{c_{i,j}}\left[\sum_{k=1}^{N_{s}}{\left(V^{\pi }\left(s_{k}\right)\right)}^{2}T\left(s_{i},a_{j},s_{k}\right)\left(1-T\left(s_{i},a_{j},s_{k}\right)\right)\right.\;\left.-2\sum_{k=1}^{N_{s}}\sum_{k^{\prime }\neq k}V^{\pi }\left(s_{k}\right)V^{\pi }\left(s_{k^{\prime }}\right)T\left(s_{i},a_{j},s_{k}\right)T\left(s_{i},a_{j},s_{k^{\prime }}\right)\right]$$

$T\left(s_{i},a_{j},s_{k}\right)$ is the true transition probability. We repeated our empirical experiments choosing the value of $\epsilon_{i,j}$ that minimizes the MSE, $\text{MSE}\left({\hat{Q}}_{\epsilon }^{\pi }\left(s_{i},a_{j}\right)\right)={\text{Bias}}^{2}\left({\hat{Q}}_{\epsilon }^{\pi }\left(s_{i},a_{j}\right)\right)+\text{Var}\left({\hat{Q}}_{\epsilon }^{\pi }\left(s_{i},a_{j}\right)\right)$, using the true value of T to compute an upper bound on the performance of the method. Results were similar to those using $\epsilon_{i,j}$ as calculated in Sec. D.2.

## Appendix E.

### Model-Free Additional Empirical Results

The performance of our regularization methods reflect the fact that model-based methods generally perform better than model-free methods in low-data settings. As we increase the size of the data set used to compute the optimal policy, demonstrated in Fig. 11, the performance of the model-free methods approach that of the model-based methods. However, at the point where there is sufficient data for the model-free methods to perform well, regularization is no longer needed.

Fig. 12 compares our methods (again without estimation error), on the other two environments, showing that performance is worse for the model-free methods compared with model-based in all cases. The case of the Random Chain environment is most promising. However, even for this environment, the lower loss in comparison to global parameter methods was not replicated when using estimated values of Q to compute $\epsilon_{i.j}^{\text{*}}$, as demonstrated in Fig. 13.

## Appendix F.

### Continuous State Space State-Action-Specific Regularization

We model the expected next state given current state and action using the Nadaraya-Watson kernel estimator,

$${\hat{T}}_{NW}\left(s,a_{k}\right)=\frac{\sum_{i=1}^{D}K_{h}\left(s-s_{i}\right)s_{i}^{'}}{\sum_{i}K_{h}\left(s-s_{i}\right)}$$

for D data tuples ${\left\{s,a_{k},s^{\prime }\right\}}_{i=1}^{D}$ for given action $a=a_{k}$. $K_{h}$ is a kernel function of lengthscale h.

Our regularized estimate of next state is:

$${\hat{T}}_{\epsilon }\left(s,a_{k}\right)=\left(1-\epsilon \right){\hat{T}}_{NW}\left(s,a_{k}\right)+\epsilon T_{reg}$$

where $T_{reg}$ is the mean of the chosen regularization transition distribution. We choose the value of ϵ that minimizes the MSE of ${\hat{T}}_{\epsilon }\left(s,a_{k}\right)$ separately for any state-action pair. To estimate the bias and variance of ${\hat{T}}_{\epsilon }\left(s,a_{k}\right)$, we use the estimates of bias and variance implied by the approximate kernel regression confidence bounds provided in Wasserman (2004).

### Bias

The approximate kernel regression confidence bounds are symmetric, implying zero bias, so the bias in ${\hat{T}}_{\epsilon }\left(s,a_{k}\right)$ comes from the second term only.

$$\text{Bias}\left({\hat{T}}_{\epsilon }\left(s,a_{k}\right)\right)\approx \epsilon \left(T_{reg}-{\hat{T}}_{NW}\left(s,a_{k}\right)\right)$$

### Variance

The approximate kernel regression confidence bounds use a standard error of

$$\hat{se}\left(s,a_{k}\right)=\hat{\sigma }\sqrt{\sum_{i=1}^{D}{\left[\frac{K_{h}\left(s-s_{i}\right)}{\sum_{j=1}^{D}K_{h}\left(s-s_{j}\right)}\right]}^{2}}$$

Where ${\hat{\sigma }}^{2}=\frac{1}{2\left(n-1\right)}\sum_{d=1}^{D-1}{\left(s_{d+1}^{'}-s_{d}^{'}\right)}^{2}$ and the data for each action is ordered by the values of $\left\{s_{d}\right\}$.

$T_{reg}$ is independent of the data, so the second term does not contribute to the variance. This implies the following variance of our regularized estimator:

$$\text{Variance}\left({\hat{T}}_{\epsilon }\left(s,a_{k}\right)\right)\approx {\left(1-\epsilon \right)}^{2}{\hat{se}}^{2}\left(s,a_{k}\right)$$

### MSE

$$\text{MSE}\left({\hat{T}}_{\epsilon }\left(s,a_{k}\right)\right)\approx f\left(\epsilon \right)=\epsilon^{2}{\left(T_{reg}-{\hat{T}}_{NW}\left(s,a_{k}\right)\right)}^{2}+{\left(1-\epsilon \right)}^{2}{\hat{se}}^{2}\left(s,a_{k}\right)\;\;f^{\prime }\left(\epsilon \right)=2\epsilon {\left(T_{\text{reg }}-{\hat{T}}_{NW}\left(s,a_{k}\right)\right)}^{2}-2\left(1-\epsilon \right){\hat{se}}^{2}\left(s,a_{k}\right)=0\;\;0=\epsilon 2{\left(T_{reg}-{\hat{T}}_{NW}\left(s,a_{k}\right)\right)}^{2}-2{\hat{se}}^{2}\left(s,a_{k}\right)+2\epsilon {\hat{se}}^{2}\left(s,a_{k}\right)\;\;{\hat{se}}^{2}\left(s,a_{k}\right)=\epsilon \left[{\left(T_{\text{reg }}-{\hat{T}}_{NW}\left(s,a_{k}\right)\right)}^{2}+{\hat{se}}^{2}\left(s,a_{k}\right)\right]\;\;\epsilon =\frac{{\hat{se}}^{2}\left(s,a_{k}\right)}{{\left(T_{reg}-{\hat{T}}_{NW}\left(s,a_{k}\right)\right)}^{2}+{\hat{se}}^{2}\left(s,a_{k}\right)}$$

## Appendix G.

### Continuous State Space Empirical Example Details

The empirical example used to demonstrate the continuous state-action specific regularization algorithm in Sec. 7.2 is a continuous-state version of the River Swim environment described in Sec. 6.1.

### State Space

We consider a one-dimensional state space, $s\in \left[0,1\right]$.

### Discount Factor

We evaluate all policies using a true discount factor of γ=0.99.

### Reward Function

We define the reward function $R\left(s\right)={\left(s-0.5\right)}^{2}+s{\left(s-.5\right)}^{3}$. As illustrated in Fig. 14, this results in a smaller positive reward near the lower end of the state space and a large postive reward at the upper end of the state space.

### Actions

There are two discrete actions. Action 0 moves the agent “upstream” towards the larger reward with greater stochasticity. Action 1 moves the agent “downstream” towards the smaller reward with less stochasticity. The transition distributions for each action are described below.

### Transition Function

$$T_{A0}\left(s,\text{noise\_level }\right)=\left\{\begin{array}{l} s^{\prime }∼\text{Unif}\left[0,1\right]\text{ with probability }0.50\\ s^{\prime }=s+0.20+\text{noise\_level}\times \text{Unif}\left[0,1\right]\text{ with probability }0.45\\ s^{\prime }=s-0.20+\text{noise\_level}\times \text{Unif}\left[0,1\right]\text{ with probability }0.05 \end{array}\right.$$

$$T_{A1}\left(s,\text{noise\_level }\right)=\left\{\begin{array}{l} s^{\prime }∼\text{Unif}\left[0,1\right]\text{ with probability }0.25\\ s^{\prime }=s-0.20+\text{noise\_level}\times \text{Unif}\left[0,1\right]\text{ with probability }0.75 \end{array}\right.$$

#### G. 1 Extensions to Online RL

The examples in the paper apply our ideas to offline RL, however it is possible to apply the same concepts to online RL. For example, we can modify the Q-learning algorithm to incorporate a weighted average update rule at each step, as demonstrated in Algorithm 3 and associated results in Fig. 15.

## Appendix H.

### Code Repository

Python code to reproduce the results in this paper is available at at: https://github.com/dtak/rethinking_discount_reg_public.

$$\begin{array}{l} \bar{\underline{\mathrm{Algorithm}3\text{ Q-learning with State-Action-Specific Regularization}}}\\ \text{ Parameters: step size }\alpha \in (0,1],\text{ regularization matrix }T_{reg}(s,a)\\ \text{ Initialize }Q(s,a)=0\forall (s,a)\\ \;\mathrm{for}\;e=1\;\mathrm{to}\;[\text{number of episodes}]\;\mathrm{do}\\ \text{ Choose initial state }s\text{ randomly}.\\ \;\mathrm{while}\text{ step\_counter}<\left[\text{steps per episode}\right]\;\mathrm{do}\\ \text{ Choose action }a\text{ from policy based on }Q(s,a)\;(\text{e.g.epsilon-greedy based on current Q})\\ \text{ Calculate }\epsilon^{*}\text{ from Eq.7}\\ \text{ Draw }x\sim Bernoulli(\left.\epsilon^{*}\right)\\ \;\mathrm{if}\;x=1\;\mathrm{then}\\ \text{ Draw simulated next step }s_{sim}^{\prime }\text{ from }T_{reg}(s,a)\\ \text{ Update Q-function using }s_{sim}^{\prime }:\\ \;Q(s,a)\leftarrow Q(s,a)+\alpha \left[r(s,a)+\gamma \max_{a}Q\left(s_{sim}^{\prime },A\right)-Q(s,a)\right]\\ \;\mathrm{else}\;\mathrm{if}\;x=0\;\mathrm{then}\\ \text{ Agent take saction }a,\text{ observes next state }s^{\prime }\\ \;Q(s,a)\leftarrow Q(s,a)+\alpha \left[r(s,a)+\gamma \max_{a}Q\left(s^{\prime },A\right)-Q(s,a)\right]\\ \text{ step\_counter}+=1\\ \;s\leftarrow s^{\prime }\\ \;\mathrm{end}\;\mathrm{if}\\ \;\mathrm{end}\;\mathrm{while}\\ \underline{\;\mathrm{end}\;\mathrm{for}} \end{array}$$
